# Design, Synthesis and Biological Assessment of *N*′-(2-Oxoindolin-3-ylidene)-6-methylimidazo[2,1-*b*]thiazole-5-carbohydrazides as Potential Anti-Proliferative Agents toward MCF-7 Breast Cancer

**DOI:** 10.3390/ph17020216

**Published:** 2024-02-07

**Authors:** Najla A. Alshaye, Mohamed K. Elgohary, Mahmoud S. Elkotamy, Hatem A. Abdel-Aziz

**Affiliations:** 1 Department of Chemistry, College of Science, Princess Nourah bint Abdulrahman University, P.O. Box 84428, Riyadh 11671, Saudi Arabia; naalshaye@pnu.edu.sa; 2 Pharmaceutical Chemistry Department, Faculty of Pharmacy, Egyptian-Russian University, Cairo 11829, Egypt; mahmoud-elkotamy@eru.edu.eg; 3 Applied Organic Chemistry Department, National Research Center, Dokki, Cairo 12622, Egypt

**Keywords:** imidazo[2,1-*b*]thiazole, isatin, MCF-7 cell line, VGEFR-2, PCR, cell cycle, apoptosis, molecular docking, ADME

## Abstract

Breast cancer is a serious threat to the health and lives of women. Two novel series of *N*′-(2-oxoindolin-3-ylidene)-6-methylimidazo[2,1-*b*]thiazole-5-carbohydrazides and 1-(aryl)-3-(6-methylimidazo[2,1-*b*]thiazol-5-yl)ureas were designed, synthesized and investigated for their anticancer efficacy against the MCF-7 breast cell line. Three compounds of the first series showed potent activity toward MCF-7 with IC_50_ in the range 8.38–11.67 µM, respectively, as compared to Sorafenib (IC_50_ = 7.55 µM). *N*′-(1-butyl-2-oxoindolin-3-ylidene)-6-methylimidazo[2,1-*b*]thiazole-5-carbohydrazide inhibited VEGFR-2 with IC_50_ = 0.33 µM when compared with Sorafenib (IC_50_ = 0.09 µM). Furthermore, this compound was introduced to PCR assessment, where it increased Bax, caspase 8, caspase 9 and cytochrome C levels by 4.337-, 2.727-, 4.947- and 2.420-fold, respectively, while it decreased levels of Bcl-2, as the anti-apoptotic gene, by 0.359-fold when compared to the untreated control MCF-7. This compound was also arrested in the G2/M phase by 27.07%, compared with 11.31% for the control MCF-7. Furthermore, it induced early and late apoptosis in MCF-7. In addition, a molecular docking study in the VEGFR-2 active site was performed to assess the binding profile for the most active compounds. Moreover, ADME parameters of the targeted compounds were also evaluated.

## 1. Introduction

Cancer is one of the most lethal diseases, and it is rapidly evolving into one of the world’s most serious health challenges. Furthermore, the unfavorable side effects of classic non-selective chemotherapies, as well as the growth of resistance to existing chemotherapy medications, make the search for more selective novel cancer fighters a top goal. However, it is difficult to design a drug that prevents the production of aberrant cells with no influence on the exerting of normal cells [1,2]. Pathological angiogenesis, on the flip side, occurs not just in the growth of tumors but additionally in a variety of non-neoplastic conditions [3]. Angiogenesis is also suggested to be linked to a variety of disorders, including cancer [4,5]. The inhibition of angiogenesis may decrease the tumor development because it can slow tumor growth without creating significant negative or resistant effects over time [6]. The VEGF (vascular endothelial growth factor) family members are well-known angiogenic agents that govern blood and lymphatic vessel development and balance in both normal and pathological angiogenesis.

VEGF-A assumes a crucial role in directing vascular development and angiogenesis by interacting with VEGFR-2. The nuanced functions of VEGFR-2 in blood vessel development are supported by adaptor proteins. In the realm of cancer, the secretion of VEGF by cancer cells is instrumental in activating the VEGFR-2 pathway in neighboring endothelial cells, thereby participating in the phenomenon of cancer-related angiogenesis. Notably, the activation of VEGFR-2 signaling has been discerned in breast cancer cells [7,8,9].

**Figure 1:**
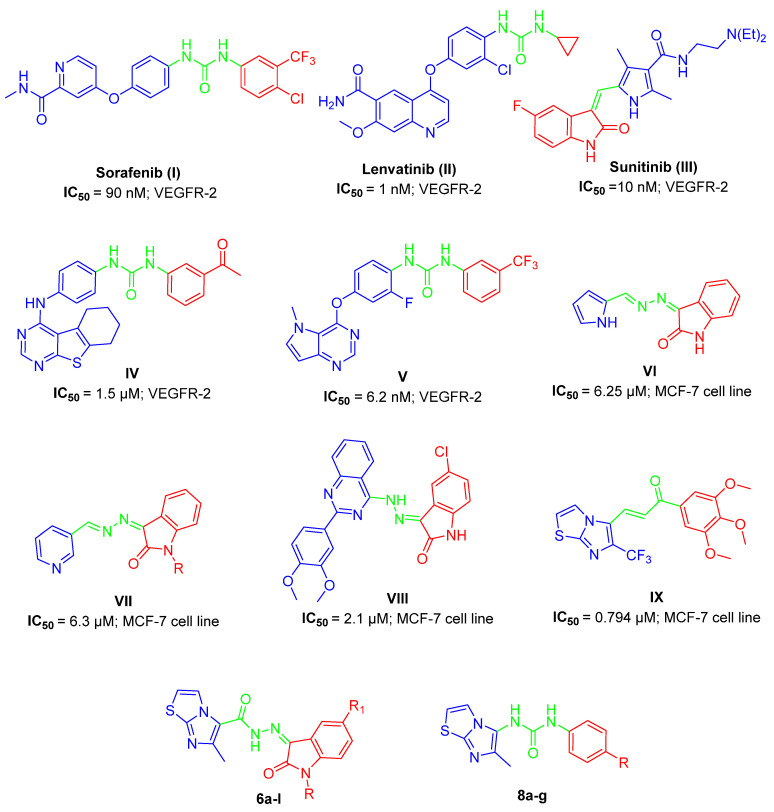
Structure of clinically approved VEGFR-2 inhibitors and some reported anticancer agents (**I**–**IX**) with their corresponding IC_50_ values, in addition to our targeted hybrids **6a**–**l** and **8a**–**g**.

Consequently, VEGFR has been extensively utilized in cancer therapy [10]. VEGF ligands bind to three different tyrosine kinases (VEGFR-TK): FLT-1 (FLT1), FLT-2 (KDR/FLK1) and FLT-4 (FLT4) [11]. VEGFR-2 is activated by the unique binding of VEGF generated by endothelial cells [12] and many malignancies have VEGFR-2 amplification [13]. Series of VEGFR-2 inhibitors have recently been approved for the management of numerous cancers such as Sorafenib (**I**) [14], Lenvatinib (**II**) [15] and Sunitinib (**III**) [16] with IC_50_= 90, 1 and 10 nM, respectively, as well as urea-based heterocycles **IV** (IC_50_= 1.5 µM) [17] and **V** (IC_50_= 6.2 nM) [18] (Figure 1).

The combinations through the hydrazine group, as a linker between position 3 of isatin (indoline-2,3-dione) and certain heterocycles such as pyrrole, pyridine or quinazoline in compounds **VI** [19], **VII** [20] and **VIII** [21], produce cytotoxic agents against the MCF-7 cancerous cell line with IC_50_ = 6.25, 6.3 and 2.1 µM, respectively (Figure 1).

A new class of imidazo[2,1-*b*]thiazoles were developed for anticancer activity and they showed potential activity toward MCF-7 cell line and elicited apoptotic properties such as caspase-9 upregulation; for example, imidazo[2,1-*b*]thiazole derivatives **IX** showed IC_50_ equal to 0.794 µM on the MCF-7 cell line and were further used for comprehensive biological investigations [22] (Figure 1).

As illustrated in Figure 1, isatin in Sunitinib (**III**) and lead compounds **VI**–**VIII**, in addition to the imidazo[2,1-*b*]thiazole system in the potent anticancer compound **IX,** are important in the synthesis of potent VEGFR-2 inhibitors and/or MCF-7 anticancer agents. On the other hand, the hydrazine linker in compounds **VI**–**VIII** and urea bridge in Sorafenib (**I**), Lenvatinib (**II**), **IV** and **V** are critical for the same purpose.

In continuation to our research, which deals with the discovery of novel potent anticancer agents [23,24,25,26,27,28], herein we utilized the fragment linking strategy to construct novel anti-proliferative agents for MCF-7 breast cancer therapy and evaluated their effectiveness on VEGFR enzyme inhibition.

**Figure 2:**
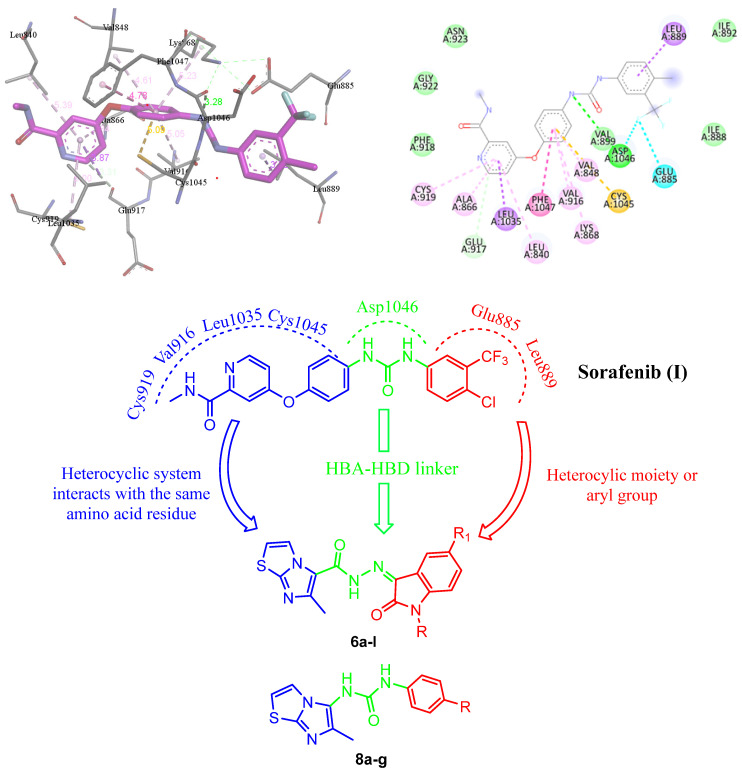
Docking of Sorafenib (**I**) with VEGFR-2 binding site and the assumed possible modifications of the targeted structures **6a**–**l** and **8a**–**g**.

The Sorafenib-VEGFR-2 crystal structure showed the role of the pyridine ring of Sorafenib (**I**) with certain amino acids in the VEGFR-2 hydrophobic pocket [29,30] (Figure 2). However, it is important to understand the SAR of synthetic VEGFR-2 kinase inhibitors [31], which may be a critical factor in determining the efficiency of the agent and its tolerability.

The binding mode of VEGFR-2 inhibitors is characterized by a “head” component that binds to the hinge region and possesses crucial H-bond donor and/or acceptor capabilities for interacting with Cys919. Additionally, they have a “linker” spanning three to four chemical bonds with H-bonding moiety to establish the interactions with Asp1046. Furthermore, they have a “tail” segment containing a hydrophobic moiety that occupies the allosteric hydrophobic back pocket [32,33,34].

In this context, we developed two sets of *N*′-(2-oxoindolin-3-ylidene)-6-methylimidazo[2,1-*b*]thiazole-5-carbohydrazides **6a**–**l** and 1-(aryl)-3-(6-methylimidazo[2,1-*b*]thiazol-5-yl)ureas **8a**–**g** (Figure 1 and Figure 2). Regarding the binding pocket of Sorafenib (**I**), the pyridine residue was specifically linked with Leu1035, Phe1047, Val848, Val916 and Cys1045 through various forms of interactions in the hinge region, whereas the H interaction was preserved via the urea linker with Asp1046. Furthermore, Leu889 and Glu885 maintained the link between the substituted phenyl ring through distinct interactions (Figure 2). Therefore, the aimed structures **6a**–**l** were designed according to the fragment combination approach, as follows: the 6-methylimidazo[2,1-*b*]thiazole system replaced the 4-phenoxypyridine moiety in the left side of Sorafenib (**I**), whereas the isatin moiety with several substitution patterns in position 1 and/or 5 replaced the aryl group of the right side of Sorafenib (**I**) with a carbohydrazide linker (H-bond acceptor–H-bond donor linker) similar to the urea bridge in Sorafenib (**I**) in order to obtain an activity similar or higher to that of Sorafenib (**I**) (Figure 2). We also followed the same approach to design urea derivatives **8a**–**g** (Figure 2). The latter two sets were synthesized and evaluated toward MCF-7 cell cytotoxicity. The most potent compounds were introduced to examine their activity toward VEGFR-2, PCR assessment, apoptosis and cell cycle analysis.

## 2. Results and Discussion

### 2.1. Chemistry

**Scheme 1:**
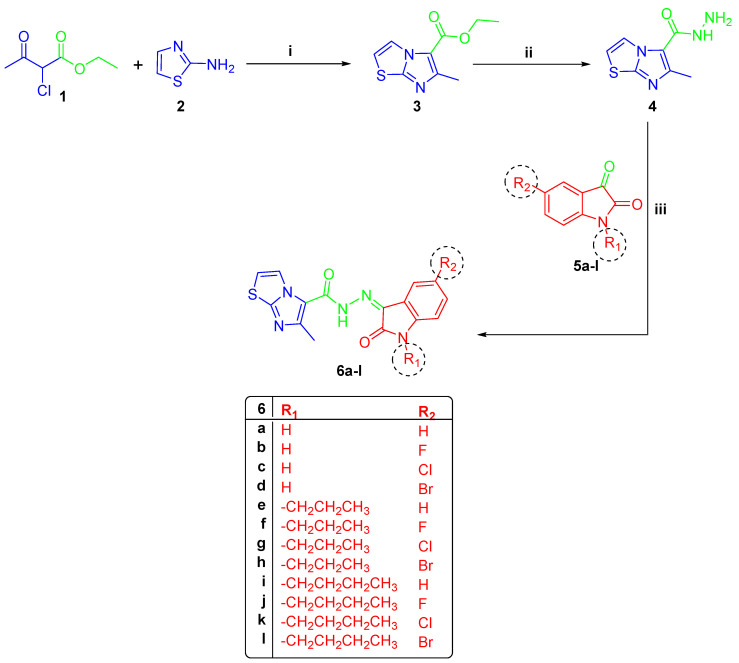
(**i**) 1,2-dimethoxyethane, ref., 6 h; (**ii**) N_2_H_4_·H_2_O/ethanol, ref., 3 h; (**iii**) EtOH/glacial acetic acid, reflux 4 h.

The reaction of 2-chloroethylacetoacetate (**1**) with 2-aminothiazole (**2**), in 1,2-dimethoxyethane at 90 °C, yielded the key ester **3**. Under reflux circumstances, the latter ester was transformed into acid hydrazide **4** using 99% hydrazine hydrate [29]. Subsequently, compound **4** was reacted with several substituted indoline-2,3-dione derivatives **5a**–**l** to yield the target 6-methyl-*N*′-(2-oxo-subsituted/unsubstituted-indolin-3-ylidene)imidazo[2,1-*b*]thiazole-5-carbohydrazides **6a**–**l** (Scheme 1). The ^1^H NMR data of compounds **6a**–**d** revealed three distinct signals, one around 2.65 ppm for CH_3_ of imidazo[2,1-*b*]thiazole and two D_2_O exchangeable signals around 11.30 and 13.26 ppm for isatin NH and carbohydrazide NH, respectively. Compounds **6e**–**h** with *n*-propyl substitution in position 1 showed three aliphatic signals of the -CH_2_-CH_2_-CH_3_ group around 0.90, 1.55–1.70 and 3.70 ppm, in addition to a singlet CH_3_ of imidazo[2,1-*b*]thiazole around 2.75 ppm. They also showed a D_2_O exchangeable hydrazide NH signal around 13.20 ppm. Furthermore, butyl-substituted compounds **6i**–**l** were characterized with five aliphatic distinguishing signals arising in the same highlights as prior propyl compounds with an extra multiplet peak of CH_2,_ in addition to the signal of imidazo[2,1-*b*]thiazole CH_3_. Concerning ^13^C NMR, spectra of **6a**–**l** exhibited distinct signal around 16.45 ppm for the CH_3_ group of imidazothiazole moiety. Moreover, ^13^C NMR of **6e**–**h** revealed three signals around 11.50, 28.82 and 42.22 ppm for the *n*-propyl group; on the other hand, compounds **6i**–**l** revealed four separate signals around 13.91, 20.00, 30.30 and 40.80 ppm for *n*-butyl substitution (Figures S4–S19, Supplementary Data).

**Scheme 2:**
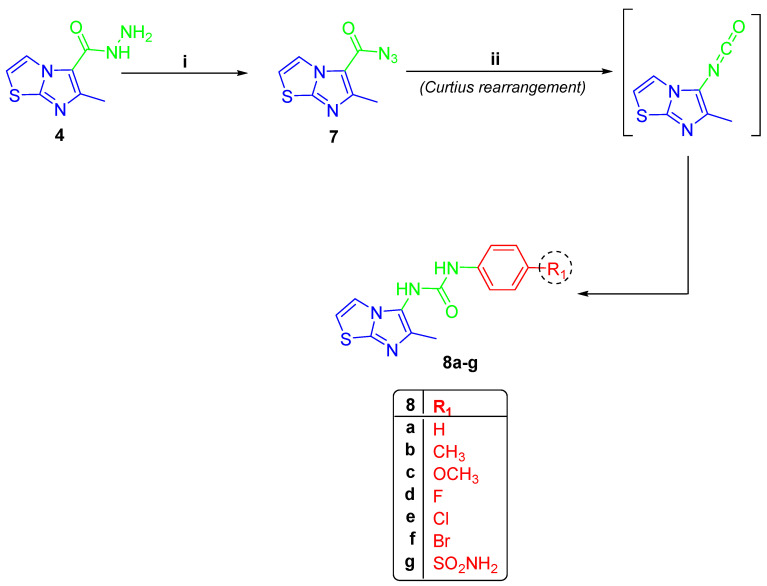
(**i**) NaNO_2_/HCl/ H_2_O, stirring 3 h; (**ii**) dioxane/diff. anilines, reflux 8 h.

On the other hand, compound **4** was subjected to a *Citrous* rearrangement process by its reaction with NaNO_2_ and HCl to obtain azide **7** [30] which was then refluxed in dioxane with various anilines to yield urea derivatives **8a**–**g** (Scheme 2). The ^1^H NMR spectra of compounds **8a**–**g** revealed three signals, one around 2.12 ppm for CH_3_ protons and the remaining two signals around 8.16 ppm and 9.02 ppm for two D_2_O exchangeable NH protons of the urea linker. Compound **8g** was characterized with an additional D_2_O exchangeable signal of sulphonamide NH_2_ at 7.17 ppm. The existence of two separate signals around 13.37 ppm and 154.20 ppm for the CH_3_ and carbonyl groups was seen in ^13^C NMR spectra of **8a**–**g**. The ^1^H NMR of compounds **8b** and **8c** showed the signals of -CH_3_ and -OCH_3_ protons, respectively, and the appearance of their carbon signals in ^13^C NMR (Figures S20–S33, Supplementary Data).

### 2.2. Anticancer Activity

#### 2.2.1. Cytotoxicity of the Designed Compounds **6a**–**l** and **8a**–**g** on MCF-7 and MCF-10A

The anti-cancer potency of **6a**–**l** and **8a**–**g** was investigated towards the MCF-7 breast cell line using MTT assay and their IC_50_ values were determined, compared to Sorafenib (**I**) [35]. The majority of the compounds **6a**–**l** and **8a**–**g** have a wide range of activity against the MCF-7 cell line, with IC_50_ ranging from 23.97 ± 0.67 µM for **6a** to 114.61 ± 4.99 µM for **8b** comparing with Sorafenib (**I**) (IC_50_ = 7.55 ± 0.40 µM) (Table 1). On the other side, compounds **6b, 6i** and **6j** showed potent anticancer activity toward MCF-7 cell lines, with IC_50_ = 11.50 ± 0.52, 8.38 ± 0.62 and 11.67 ± 0.52 µM, respectively, as compared to Sorafenib (**I**) (IC_50_ = 7.55 ± 0.40 µM) (Table 1).

On the other hand, compounds **6b, 6i** and **6j** revealed non-cytotoxic activity toward MCF-10A due to its non-tumorigenic origin [36], where their IC_50_ = 125.26 ± 2.7, 54.63 ± 1.29 and 47.73 ± 0.53 µM, when compared to Sorafenib (IC_50_ = 22.35 ± 1.29 µM) (Table 2).

#### 2.2.2. SARs of Compounds **6a**–**l** and **8a**–**g** on MCF-7 Cell Line

SARs assessments of the aforementioned data of compounds **6a**–**l** revealed that the linking between the imidazo[2,1-*b*]thiazole system and indoline-2,3-dione via the hydrazide bridge showed a wide range of activity as **6c**, **6e**, **6f**, **6g**, **6h**, **6k** and **6l** have weak antiproliferative activity, with an IC_50_ range of 43.10 ± 3.69–82.55 ± 2.75 µM in comparison to Sorafenib (**I**) (IC_50_ = 7.55 ± 0.40 µM), while compounds **6a** (IC_50_ = 23.97 ± 0.67 µM) and **6d** (IC_50_ = 22.80 ± 1.38 µM) revealed moderate anti-proliferative activity. Regarding **6b**, **6i** and **6j**, they showed the highest anticancer activity with an IC_50_ range = 8.38 ± 0.62–11.67 ± 0.52 µM. *N*′-(1-butyl-2-oxoindolin-3-ylidene)-6-methylimidazo[2,1-*b*]thiazole-5-carbohydrazide (**6i**) with *n*-butyl substitution (R = *n*-butyl) in position 1 of isatin moiety exhibited the highest anticancer activity (IC_50_ = 8.38 ± 0.62 µM), followed by **6b** with fluoro-substitution (R_1_ = F) in position 5 of isatin moiety, with an IC_50_ = 11.50 ± 0.52 µM, whereas compound **6j** with *n*-butyl substitution (R_1_ = *n*-butyl) and fluoro-substitution (R_2_ = F) in positions 1 and 5 of isatin moiety, respectively, had an IC_50_ range = 11.67 ± 0.52 µM (Table 1). From the latter data, the variation in substitutions in positions 1 and 5 of isatin moiety plays an important role in the anticancer activity of hybrids **6a**–**l**; for example, the impact of F is higher than Cl or Br, whereas the effect of *n*-butyl substitution is higher than that of *n*-propyl. On the other side, imidazo[2,1-*b*]thiazoles with arylurea branch compounds **8a**–**g** showed anticancer activities lower than those of **6a**–**l**.

#### 2.2.3. VGEFR-2 Inhibitory Activity (IC_50_) for Compounds **6b**, **6i** and **6j**

The VEGFR-2 inhibitory activity for compounds **6b**, **6i** and **6j** was investigated and the results in IC_50_ computed from the concentration–inhibition response curve compared to Sorafenib (**I**) are summarized in Table 3. The VEGFR-2 inhibitory effects of **6b**, **6i** and **6j**, respectively, showed good activity, with an IC_50s_ = 3.85 ± 0.15, 0.33 ± 0.01 and 1.51 ± 0.06 µM, respectively, when compared to Sorafenib (**I**) (IC_50_ = 0.09 ± 0.004 µM). Compound **6j** (IC_50_ = 1.51 ± 0.06 µM) displayed a significant influence on kinase activity when compared to **6b** with IC_50_ = 3.85 ± 0.15 µM, which indicated the effect of positions 1 and 5 substitutions of isatin moiety. However, compound **6j** revealed the highest VEGFR-2 inhibition activity, with IC_50_ = 0.33 ± 0.01 µM.

#### 2.2.4. PCR Assessment for Compound **6i**

The gene expression levels of apoptosis genes in MCF-7 cells were measured using RT-PCR to validate the apoptosis-inducing behaviors of the potent lead compound **6i** in MCF-7 cells. Compound **6i** increased Bax levels by 4.337-fold, caspase 8 levels by 2.727-fold, caspase 9 levels by 4.947-fold and cytochrome C levels by 2.420-fold, while it decreased the level of Bcl-2, as the anti-apoptotic gene, by 0.359-fold when compared to the untreated control MCF-7 (Table 4).

**Figure 3:**
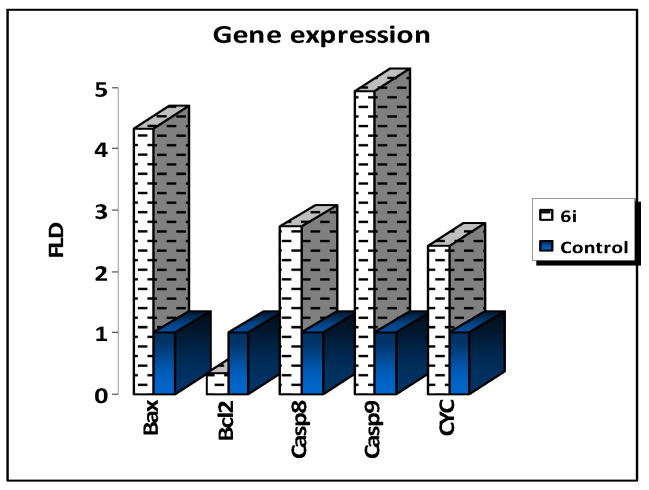
Quantitative RT-PCR results analysis of the apoptosis-related genes, cytochrome, Bax, Caspases 8, 9 and Bcl-2, in **6i**-treated MCF-7 cells. Dashed line represents the gene expression for potent **6i** and blue line represents untreated control.

The results revealed an inherent apoptotic mechanism including the activation of Bax, cytochrome C, and caspases 3 and 9. As a result, our RT-PCR findings supported prior research by demonstrating that the apoptotic pathway is characterized by the overexpression of proapoptotic genes and the downregulation of anti-apoptotic genes (Figure 3).

#### 2.2.5. Cell Cycle Analysis of Compound **6i** on MCF-7

**Figure 4:**
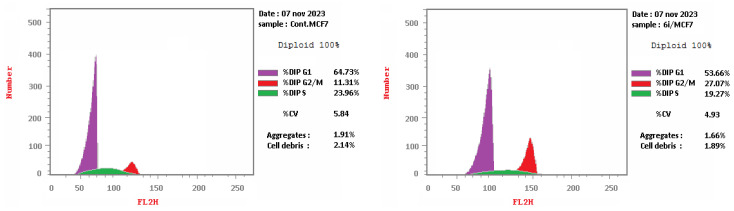
Cell cycle for cont. MCF-7 (**left**) and **6i** (**right**).

Compound **6i** arrested cells in the G2/M phase with %G2/M = 27.07, compared with %G2/M = 11.31 for the control MCF-7 (Table 5 and Figure 4).

#### 2.2.6. Apoptotic Assay for Compound **6i**

**Figure 5:**
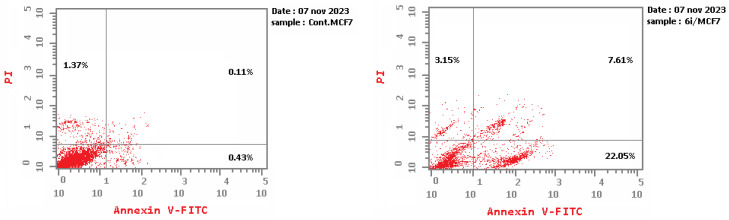
Apoptosis analysis for control MCF-7 (**left**) and **6i** (**right**).

In comparison to the control cells, the apoptotic assay employing Annexin V/PI analysis for compound **6i** generated a substantial amount of early and late apoptosis in MCF-7. Compound **6i** induced early apoptosis in MCF-7 at rate of 22.05, compared with the control MCF-7 (0.43) (Table 6 and Figure 5). Furthermore, **6i** demonstrated late apoptosis at rate 7.61 compared with the control MCF-7 (0.11).

### 2.3. Molecular Modeling Study

The computational docking method is used for approximating two fundamental concepts. The main objective is to determine the correct spot and orientation of a particular chemical pose in the binding site of the enzyme. The second concept is the calculation of docking score (the strength of protein-ligand interactions) [37]. In an attempt to explain the cytotoxic action of **6b**, **6i** and **6j**, a docking study in the VEGFR-2 active site was performed. Redocking of sorafenib (**I**) in the active site of VEGFR-2 (PDB: 4ASD) was nearly in the exact position as the co-crystalized ligand with d. s. = −10.6 kcal/mol) (Figure S1, Supplementary Data).

**Figure 6:**
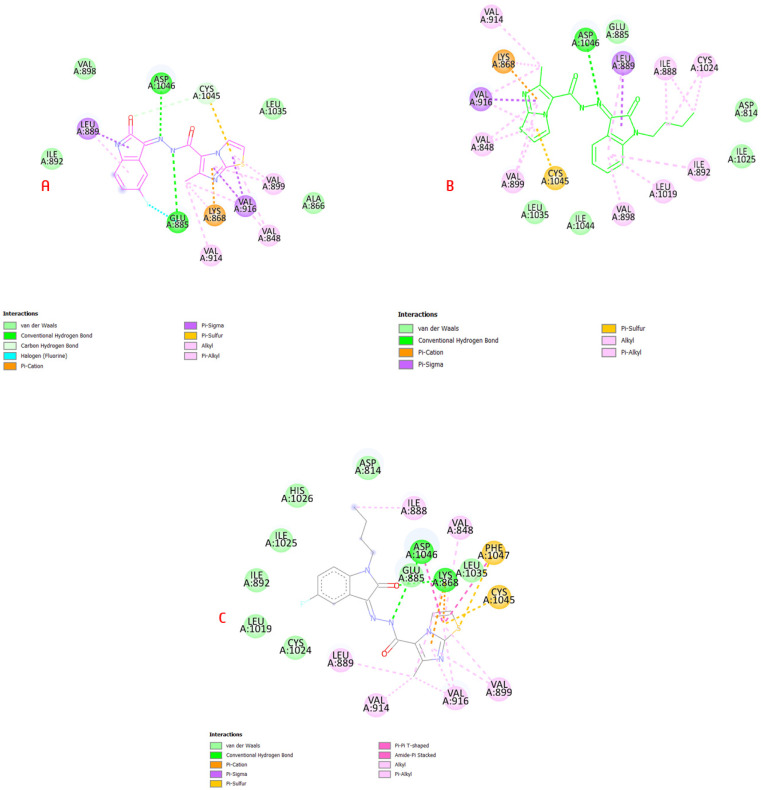
(**A**) **6b** in VEGR-2 binding site showed H-bond with Asp1046 and pi-cation interaction with Lys868; (**B**) compound **6i** revealed H-bond with Asp1046, pi-sigma interaction with Cys1045 and pi-cation interaction with Lys868; (**C**) **6j** showed H-interactions with Asp1046 and Lys868 residues (Table S1, Supplementary Data).

The docking investigation of **6b**, **6i** and **6j** in the VEGFR-2 active site revealed binding mechanisms that were similar to the lead medicine and they showed docking scores that were higher or comparable to the docked lead. The hot areas were satisfied by targeted compounds, which established H-bonds with Cys919, Glu885 and Asp1046 residues which helped to explain the substantial VEGFR-2 inhibitory effect, as shown in Figure 6.

As shown in the interactions of **6b**, **6i** and **6j** on the VEGFR-2 active site, they interacted with the same pattern of sorafenib (**I**) with a hydrogen-bond interaction with amino acid Asp1046 via the linker hydrazide moiety, while imidazothiazole bonded with Val916 and Cys1045 as the pyridine side; on the other hand, phenyl isatin bonded with Leu889 via pi-sigma as the substituted phenyl in the Sorafenib (**I**) structure (Figure S2, Supplementary Data).

### 2.4. Computational ADME Study

Three rules, at least, of the following five rules are requested for oral bioavailability: There are no more than five H-bond donors, no more than 500 Da MWt, no more than five log P, only one violation and no more than ten H-bond acceptors, according to Lipinski’s rule of five [38]. The Veber rule predicts that molecules with less than 10 rotatable bonds and at least 140.2 of polar surface area will have appropriate oral bioavailability [39]. ADME parameters of the targeted compounds **6a**–**l** and **8a**–**g** such as the proportion of human intestinal absorption and blood–brain barrier penetration were evaluated using the SWISSADME method. Their BBB penetration was expected to be minimal, which prevents these candidates from passing the BBB or having harmful effects on the central nervous system (Figure S3 and Table S2, Supplementary Data).

## 3. Materials and Methods

### 3.1. Chemistry

The specifications of instruments used in the chemistry part are listed in the Supplementary Data, page 42.

#### 3.1.1. Preparation of Carbohydrazide **4**

A mixture of 2-aminothiazole (**1**) (10.0 g, 100 mmol) and ethyl 2-chloroacetoacetate (**2**) (16.5 g, 100 mmol) was refluxed for 6 h in 1,2-dimethoxyethane (50 mL). After evaporation of the solvent, off-white precipitate was formed and then recrystallized from EtOH to afford compound **3**. The latter ester **3** (2.10 g, 10 mmol) was treated with hydrazine hydrate (99%, 2 mL) for 4 h in refluxed EtOH (50 mL). The solvent was evaporated to obtain white precipitate, which then recrystallized from EtOH to give carbohydrazide **4** [40].

#### 3.1.2. Synthesis of Hybrids **6a**–**l**

A mixture of carbohydrazide **4** (0.196 g, 1.0 mmol) and indoline-2,3-dione derivatives **5a**–**l** (1.0 mmol) in EtOH (30 mL) and AcOH (0.3 mL) was refluxed for 4 h. Crystallization of the resulting solid from EtOH/DMF gave compounds **6a**–**l**, respectively.

##### 6-Methyl-*N*′-(2-oxoindolin-3-ylidene)imidazo[2,1-*b*]thiazole-5-carbohydrazide (**6a**)

Yield = 68%; m.p. = >300 °C; IR: 1697 (>C=O), 3263 (>N-H), 3444 (>N-H); ^1^H NMR: 2.65 (s, 3H, -CH_3_), 6.92 (d, *J* = 8.1 Hz, 1H, Ar-H), 7.07 (d, *J* = 8.1 Hz, 1H, Ar-H), 7.31–7.36 (m, 1H, Ar-H), 7.41 (d, *J* = 4.5 Hz, 1H, Ar-H), 7.53 (d, *J* = 7.5 Hz, 1H, Ar-H), 8.21 (d, *J* = 4.5 Hz, 1H, Ar-H), 11.11 (s, 1H, >N-H), 13.26 (s, 1H, >N-H); ^13^C NMR: 16.88 (-CH_3_), 111.73, 115.41, 117.66, 120.61, 121.22, 121.74, 132.04, 137.52, 142.82, 148.80, 152.63, 157.08 (>C=O), 163.16 (>C=O); Anal. for C_15_H_11_N_5_O_2_S (325.35): calcd.: C, 55.38; H, 3.41; N, 21.53; found: C, 55.50; H, 3.47; N, 21.37.

##### *N*′-(5-Fluoro-2-oxoindolin-3-ylidene)-6-methylimidazo[2,1-*b*]thiazole-5-carbohydrazide (**6b**)

Yield = 74%; m.p. = >300 °C; IR: 1710 (>C=O), 3294 (>N-H), 3420 (>N-H); ^1^H NMR: 2.65 (s, 3H, -CH_3_), 6.93 (q, *J* = 4.0 Hz, 1H, Ar-H), 7.15–7.19 (m, 1H, Ar-H), 7.33 (dd, *J* = 8.1 Hz, 1H, Ar-H), 7.42 (d, *J* = 4.1 Hz, 1H, Ar-H), 8.22 (d, *J* = 4.0 Hz, 1H, Ar-H), 11.14 (s, 1H, >N-H), 13.28 (s, 1H, >N-H); ^13^C NMR: 16.89 (-CH_3_), 40.73, 108.34, 112.85, 115.54 (2C), 117.59, 118.29, 121.75 (2C), 139.05, 149.06, 152.81, 157.01 (>C=O), 163.29 (>C=O); Anal. for C_15_H_10_FN_5_O_2_S (343.34): calcd.: C, 52.47; H, 2.94; N, 20.40; found: C, 52.21; H, 2.99; N, 20.53.

##### *N*′-(5-Chloro-2-oxoindolin-3-ylidene)-6-methylimidazo[2,1-*b*]thiazole-5-carbohydrazide (**6c**)

Yield = 67%; m.p. = 292–293 °C; IR: 1705 (>C=O), 3124 (>N-H), 3367 (>N-H); ^1^H NMR: 2.68 (s, 3H, -CH_3_), 6.95 (d, *J* = 8.5 Hz, 1H, Ar-H), 7.39 (d, *J* = 4.0 Hz, 1H, Ar-H), 7.48 (d, *J* = 4.0 Hz, 1H, Ar-H), 7.51 (m, 1H, Ar-H), 8.26 (d, *J* = 4.4 Hz, 1H, Ar-H),11.38 (s, 1H, >N-H), 13.26 (s, 1H, >N-H); ^13^C NMR: 16.91 (-CH_3_), 113.25, 115.53, 117.56, 120.67, 121.75, 122.31, 127.42, 131.39, 136.48, 141.44, 149.03, 152.80, 156.92 (>C=O), 162.94 (>C=O); Anal. for C_15_H_10_ClN_5_O_2_S (359.79): calcd.: C, 50.08; H, 2.80; N, 19.47; found: C, 49.91; H, 3.01; N, 19.50.

##### *N*′-(5-Bromo-2-oxoindolin-3-ylidene)-6-methylimidazo[2,1-*b*]thiazole-5-carbohydrazide (**6d**)

Yield = 66%; m.p. = 296–297 °C; IR: 1705 (>C=O), 3116 (>N-H), 3383 (>N-H); ^1^H NMR: 2.62 (s, 3H, -CH_3_), 6.85 (s, 1H, Ar-H), 7.42–7.46 (m, 2H, Ar-Hs), 7.56 (s, 1H, Ar-H), 8.20 (s, 1H, Ar-H), 11.33 (s, 1H, >N-H), 13.20 (s, 1H, >N-H); ^13^C NMR: 13.36 (-CH_3_), 112.07 (2C), 115.59, 115.76 (2C), 118.58, 118.98, 1120.82, 136.62, 137.29, 144.84, 154.29, 156.98 (C=O), 158.87 (C=O); Anal. for C_15_H_10_BrN_5_O_2_S (404.24): calcd.: C, 44.57; H, 2.49; N, 17.33; found: C, 44.30; H, 2.36; N, 17.37.

##### 6-Methyl-*N*′-(2-oxo-1-propylindolin-3-ylidene)imidazo[2,1-*b*]thiazole-5-carbohydrazide (**6e**)

Yield = 51%; m.p. = 256–257 °C; IR: 1702 (>C=O), 3236 (>N-H); ^1^H NMR: 0.89 (t, *J* = 7.5 Hz, 3H, -CH_3_), 1.62–1.69 (m, 2H, >CH_2_), 2.67 (s, 3H, -CH_3_), 3.71 (t, *J* = 7.5 Hz, 2H, >CH_2_), 7.12–7.19 (m, 2H, Ar-Hs), 7.41–7.44 (m, 2H, Ar-Hs), 7.59 (d, *J* = 8.1 Hz, 1H, Ar-H), 8.22 (d, *J* = 4.0 Hz, 1H, Ar-H), 13.21 (s, 1H, >N-H); ^13^C NMR: 11.55 (-CH_3_), 17.03 (-CH_3_), 28.89 (>CH_2_), 41.65 (>CH_2_), 112.80, 113.01, 115.65, 121.82, 123.48, 134.33, 135.65, 142.20, 148.27, 152.71, 156.25, 161.10 (>C=O); Anal. for C_18_H_17_N_5_O_2_S (367.43): calcd.: C, 58.84; H, 4.66; N, 19.06; found: C, 59.07; H, 4.59; N, 19.20.

##### *N*′-(5-Fluoro-2-oxo-1-propylindolin-3-ylidene)-6-methylimidazo[2,1-*b*]thiazole-5-carbohydrazide (**6f**)

Yield = 66%; m.p. = 243–244 °C; IR: 1689 (>C=O), 3224 (>N-H); ^1^H NMR: 0.92 (t, *J* = 7.5 Hz, 3H, -CH_3_), 1.64–1.70 (m, 2H, >CH_2_), 2.72 (s, 3H, -CH_3_), 3.76 (t, *J* = 7.5 Hz, 2H, >CH_2_), 7.26–7.32 (m, 2H, Ar-Hs), 7.43–7.45 (m, 1H, Ar-H), 7.48 (d, *J* = 4.4 Hz, 1H, Ar-H), 8.38 (s, 1H, Ar-H), 13.21 (s, 1H, >N-H); ^13^C NMR: 11.51 (-CH_3_), 17.20 (-CH_3_), 29.90 (>CH_2_), 41.71 (>CH_2_), 113.21, 113.29, 114.50, 123.18, 125.58, 132.10, 134.23, 143.60, 149.55, 153.79, 156.63, 162.12 (>C=O); Anal. for C_18_H_16_FN_5_O_2_S (385.42): calcd.: C, 56.09; H, 4.18; N; 18.17; found: C, 55.82; H, 4.21; N; 18.30.

##### *N*′-(5-Chloro-2-oxo-1-propylindolin-3-ylidene)-6-methylimidazo[2,1-*b*]thiazole-5-carbohydrazide (**6g**)

Yield = 61%; m.p. = 254–255 °C; IR: 1693 (>C=O), 3236 (>N-H); ^1^H NMR: *E*/*Z*, 0.83, 0.87 (2t, *J* = 7.0 Hz, *J* = 7.5 Hz, 3H, -CH_3_), 1.54–1.63 (2m, 2H, >CH_2_), 2.52, 2.68 (2s, 3H, -CH_3_), 3.66, 3.72 (2t, *J* = 7.0 Hz, *J* = 7.5 Hz, 2H, >CH_2_), 7.18, 7.26 (2d, *J* = 8.5 Hz, *J* = 9.0 Hz, 1H, Ar-H), 7.39–7.45 (2d, *J* = 4.0 Hz, *J* = 4.4 Hz, 1H, Ar-H), 7.48–7.55 (m, 2H, Ar-Hs), 8.18, 8.25 (2s, 1H, Ar-H), 11.46, 13.20 (2s, 1H, >N-H); ^13^C NMR: 11.43 (-CH_3_), 16.30 (-CH_3_), 28.40 (>CH_2_), 43.51 (>CH_2_), 112.56, 113.65, 118.58, 124.29, 124.52, 132.18, 134.23, 144.64, 149.05, 154.59, 155.83, 162.23(>C=O); Anal. for C_18_H_16_ClN_5_O_2_S (401.87): calcd.: C, 53.80; H, 4.01; N, 17.43; found: C, 53.53; H, 3.84; N, 17.57.

##### *N*′-(5-Bromo-2-oxo-1-propylindolin-3-ylidene)-6-methylimidazo[2,1-*b*]thiazole-5-carbohydrazide (**6h**)

Yield = 50%; m.p. = 261–262 °C; IR: 1724 (>C=O), 3232 (>N-H); ^1^H NMR: *E*/*Z*, 0.83, 0.86 (2t, *J* = 7.0 Hz, *J* = 7.5 Hz, 3H, -CH_3_), 1.54–1.63 (2m, 2H, >CH_2_), 2.36, 2.66 (2s, 3H, -CH_3_), 3.65, 3.69 (2t, *J* = 7.0 Hz, *J* = 7.5 Hz, 2H, >CH_2_), 7.12, 7.18 (2d, *J* = 8.5 Hz, *J* = 9.0 Hz, 1H, Ar-H), 7.38, 7.44 (2d, *J* = 4.0 Hz, *J* = 4.5 Hz, 1H, Ar-H), 7.57–7.64 (m, 2H, Ar-Hs), 8.06, 8.23 (2d, *J* = 4.0 Hz, *J* = 5.0 Hz, 1H, ArH), 11.14, 13.60 (2s, D_2_O exchangeable, 1H, >N-H); ^13^C NMR: 11.58 (-CH_3_), 17.00 (-CH_3_), 28.87 (>CH_2_), 41.60 (>CH_2_), 112.75, 115.47, 115.60, 121.78, 123.28, 134.10, 135.43, 142.50, 149.17, 152.89, 156.92, 161.04 (>C=O); Anal. for C_18_H_16_BrN_5_O_2_S (446.32): calcd.: C, 48.44; H, 3.61; N, 15.69; found: C, 48.56; H, 3.53; N, 15.71.

##### *N*′-(1-Butyl-2-oxoindolin-3-ylidene)-6-methylimidazo[2,1-*b*]thiazole-5-carbohydrazide (**6i**)

Yield = 45%; m.p. = 250–251 °C; IR: 1670 (>C=O), 3240 (>N-H); ^1^H NMR: 0.92 (t, *J* = 7.2 Hz, 3H, -CH_3_), 1.35–1.39 (m, 2H, >CH_2_), 1.60–1.66 (m, 2H, >CH_2_), 2.73 (s, 3H, -CH_3_), 3.79 (t, *J* = 7.3 Hz, 2H, >CH_2_), 7.17–7.24 (m, 2H, Ar-Hs), 7.45–7.49 (m, 2H, Ar-Hs), 7.64 (d, *J*= 7.2 Hz, 1H, Ar-H), 8.34 (s, 1H, Ar-H): ^13^C NMR: 14.02 (-CH_3_), 16.97 (-CH_3_), 20.04 (>CH_2_), 29.64 (>CH_2_), 39.24 (>CH_2_), 4077, 42.23, 110.65, 115.48, 117.63, 120.02, 121.06, 121.76, 123.66, 132.02, 136.63, 143.38, 148.91, 161.32 (>C=O); Anal. for C_19_H_19_N_5_O_2_S (381.45): calcd.: C, 59.83; H, 5.02; N, 18.36; found: C, 59.67; H, 4.85; N, 18.39.

##### *N*′-(1-Butyl-5-fluoro-2-oxoindolin-3-ylidene)-6-methylimidazo[2,1-*b*]thiazole-5-carbohydrazide (**6j**)

Yield = 50%; m.p. = 237–238 °C; IR: 1670 (>C=O), 3244 (>N-H); ^1^H NMR: 0.86 (t, *J* = 7.0 Hz, 3H, -CH_3_), 1.26–1.33 (m, 2H, >CH_2_), 1.53–1.59 (m, 2H, >CH_2_), 2.66 (s, 3H, -CH_3_), 3.71 (t, *J* = 7.5 Hz, 2H, >CH_2_), 7.17–7.20 (m, 1H, Ar-H), 7.23–7.27 (m, 1H, Ar-H), 7.37 (d, *J* = 8.1 Hz, 1H, Ar-H), 7.44 (d, *J* = 4.0 Hz, 1H, Ar-H), 8.22 (d, *J* = 4.0 Hz, 1H, Ar-H), 13.20 (s, 1H, >N-H); ^13^C NMR: 14.00 (-CH_3_), 16.97 (-CH_3_), 20.01 (>CH_2_), 29.56 (>CH_2_), 39.74 (>CH_2_), 108.16, 108.36, 111.94, 115.59, 117.56, 118.07, 121.43, 136.13, 139.60, 149.14, 152.88, 156.97, 158.35, 160.26, 161.35 (>C=O); Anal. for C_19_H_18_FN_5_O_2_S (399.44): calcd.: C, 57.13; H, 4.54; N, 17.53; found: C, 57.26; H, 4.57; N, 17.80.

##### *N*′-(1-Butyl-5-chloro-2-oxoindolin-3-ylidene)-6-methylimidazo[2,1-*b*]thiazole-5-carbohydrazide (**6k**)

Yield = 67%; m.p. = 224–225 °C; IR: 1693 (>C=O), 3224 (>N-H); ^1^H NMR: 0.85 (t, *J* = 7.5 Hz, 3H, -CH_3_), 1.27–1.32 (m, 2H, >CH_2_), 1.55–1.58 (m, 2H, >CH_2_), 2.68 (s, 3H, -CH_3_), 3.74 (t, *J* = 7.5 Hz, 2H, >CH_2_), 7.24 (d, *J* = 8.1 Hz, 1H, Ar-H), 7.46–7.49 (m, 2H, Ar-Hs), 7.56 (s, 1H, Ar-H), 8.25 (d, *J* = 4.0 Hz, 1H, Ar-H), 13.19 (s, 1H, >N-H); ^13^C NMR: 14.02 (-CH_3_), 15.99 (-CH_3_), 20.00 (>CH_2_), 30.56 (>CH_2_), 40.52 (>CH_2_), 43.69, 44.96, 118.70, 121.10, 122.18, 125.52, 126.31, 127.96, 130.24, 138.18, 144.56, 149.16, 157.09, 161.58 (>C=O); Anal. for C_19_H_18_ClN_5_O_2_S (415.90): calcd.: C, 54.87; H, 4.36; N, 16.84; found: C, 55.06; H, 4.38; N, 17.00.

##### *N*′-(5-Romo-1-butyl-2-oxoindolin-3-ylidene)-6-methylimidazo[2,1-*b*]thiazole-5-carbohydrazide (**6l**)

Yield = 39%; m.p. = 237–238 °C; IR: 1693 (>C=O), 3228 (>N-H); ^1^H NMR: 0.86 (t, *J* = 7.1 Hz, 3H, -CH_3_), 1.27–1.33 (m, 2H, >CH_2_), 1.53–1.57 (m, 2H, >CH_2_), 2.66 (s, 3H, -CH_3_), 3.71 (t, *J* = 7.5 Hz, 2H, >CH_2_), 7.16 (s, 1H, Ar-H), 7.44 (d, *J* = 5.0 Hz, 1H, Ar-H), 7.58 (s, *J* = 9.5 Hz, 1H, Ar-H), 7.63 (s, 1H, Ar-H), 8.22 (s, 1H, Ar-H), 13.14 (s, 1H, >N-H); ^13^C NMR: 14.00 (-CH_3_), 16.99 (-CH_3_), 20.00 (>CH_2_), 29.56 (>CH_2_), 40.52 (>CH_2_), 40.69, 40.76, 112.72, 115.48, 115.61, 117.55, 121.78, 123.30, 134.13, 142.42, 149.18, 152.90, 156.93, 160.97 (>C=O); Anal. for C_19_H_18_BrN_5_O_2_S (460.35): calcd.: C, 49.57; H, 3.94; N, 15.21; found: C, 49.52; H, 4.08; N, 15.34.

#### 3.1.3. Synthesis of Urea Derivatives **8a**–**g**

Compound **4** was stirred for 3 h in a solution of NaNO_2_ with HCl to form the 6-methylimidazo[2,1-*b*]thiazole-5-carbonyl azide [41]. Azide **7** (0.21 g, 1 mmol) was refluxed in 1,4-dioxane (40 mL) with different anilines (1.0 mmol) for 8 h. Crystallization from EtOH for the formed solid gave the targeted urea derivatives **8a**–**g**, respectively.

##### 1-(6-Methylimidazo[2,1-*b*]thiazol-5-yl)-3-phenylurea (**8a**)

Yield = 54%; m.p. = 287–288 °C; IR: 1639 (>C=O), 3263 (>N-H), 3290 (>N-H); ^1^H NMR: 2.09 (s, 3H, -CH_3_), 6.92 (t, *J* = 7.5 Hz, 1H, Ar-H), 7.08 (d, *J* = 4.5 Hz, 1H, Ar-H), 7.22 (t, *J* = 7.4 Hz, 1H, Ar-H), 7.42 (d, *J* = 7.8 Hz, 2H, Ar-Hs), 7.53 (d, *J* = 5.0 Hz, 1H, Ar-H), 8.09 (s, 1H, NH), 8.93 (s, 1H, >N-H); ^13^C NMR: 13.36 (-CH_3_), 112.05, 118.63, 118.98 (3C), 122.48, 129.21 (2C), 137.23, 140.25, 144.81, 154.18 (>C=O); Anal. for C_13_H_12_N_4_OS (272.33): calcd.: C, 57.34; H, 4.44; N, 20.57; found: C, 57.17; H, 4.26; N, 20.79. 

##### 1-(6-Methylimidazo[2,1-*b*]thiazol-5-yl)-3-(p-tolyl)urea (**8b**)

Yield = 47%; m.p. = 282–283 °C; IR: 1701 (>C=O), 3267 (>N-H), 3360 (>N-H); ^1^H NMR: 2.14 (s, 3H, -CH_3_), 2.25 (s, 3H, -CH_3_), 7.08 (d, *J* = 8.0 Hz, 2H, Ar-Hs), 7.13 (d, *J* = 4.4 Hz, 1H, Ar-H), 7.36 (d, *J* = 7.8 Hz, 2H, Ar-Hs), 7.57 (d, *J* = 4.4 Hz, 1H, Ar-H), 8.09 (s, 1H, >N-H), 8.83 (s, 1H, >N-H); ^13^C NMR: 13.35 (-CH_3_), 20.87 (-CH_3_), 112.05, 118.70, 118.98, 119.13 (2C), 129.60 (2C), 131.32, 137.19, 137.65, 144.79, 154.20 (>C=O); Anal. for C_14_H_14_N_4_OS (286.35): calcd.: C, 58.72; H, 4.93; N, 19.57; found: C, 58.66; H, 5.10; N, 19.59.

##### 1-(4-Methoxyphenyl)-3-(6-methylimidazo[2,1-*b*]thiazol-5-yl)urea (**8c**)

Yield = 50%; m.p. = 284–285 °C; IR: 1639 (>C=O), 3140 (>N-H), 3278 (>N-H); ^1^H NMR: 2.08 (s, 3H, -CH_3_), 3.66 (s, 3H, -O-CH_3_), 6.80 (d, *J* = 8.5 Hz, 2H, ArH), 7.08 (d, *J* = 4.5 Hz, 1H, Ar-H), 7.32 (d, *J* = 8.5 Hz, 2H, Ar-Hs), 7.52 (d, *J* = 5.0 Hz, 1H, Ar-H), 8.04 (s, 1H, >N-H), 8.74 (s, 1H, >N-H); ^13^C NMR: 13.37 (-CH_3_), 55.67 (-O-CH_3_), 111.98, 114.37 (2C), 118.83, 120.6 (2C), 133.29, 137.13 144.73, 144.73, 154.37, 155.05 (>C=O); Anal. for C_14_H_14_N_4_O_2_S (302.35): calcd.: C, 55.62; H, 4.67; N, 18.53; found: C, 55.35; H, 4.69; N, 18.45.

##### 1-(4-Fluorophenyl)-3-(6-methylimidazo[2,1-*b*]thiazol-5-yl)urea (**8d**)

Yield = 56%; m.p. = 281–282 °C; IR (KBr): 1635 (>C=O), 3163 (>N-H), 3278 (>N-H); ^1^H NMR: 2.08 (s, 3H, -CH_3_), 7.04–7.10 (m, 3H, Ar-Hs), 7.42–7.44 (m, 2H, Ar-Hs), 7.54 (d, *J* = 5.0 Hz, 1H, Ar-H), 8.12 (s, 1H, >N’-H), 8.98 (s, 1H, >N-H); ^13^C NMR: 13.36 (-CH_3_), 112.07, 115.59 (2C), 115.76 (2C), 118.58, 118.98, 120.82, 136.62, 137.29, 144.84, 154.29 (>C=O); Anal. for C_13_H_11_FN_4_OS (290.32): calcd.: C, 53.78; H, 3.82; N, 19.30; found: C, 53.80; H, 4.02; N, 19.53.

##### 1-(4-Chlorophenyl)-3-(6-methylimidazo[2,1-*b*]thiazol-5-yl)urea (**8e**)

Yield = 48%; m.p. = 285–286 °C; IR: 1708 (>C=O), 3267 (>N-H), 3360 (>N-H); ^1^H NMR: 2.08 (s, 3H, -CH_3_), 7.09 (d, *J* = 4.0 Hz, 1H, Ar-H), 7.26 (d, *J* = 9.0 Hz, 2H, Ar-Hs), 7.45 (d, *J* = 8.5 Hz, 1H, Ar-H), 7.54 (d, *J* = 4.5 Hz, 1H, Ar-H), 8.19 (s, 1H, >N-H), 9.12 (s, 1H, >N-H); ^13^C NMR: 13.36 (-CH_3_), 112.10, 118.46, 118.99, 120.56 (2C), 126.00, 129.03 (2C), 137.35, 139.32, 144.89, 154.13 (>C=O); Anal. for C_13_H_11_ClN_4_OS (306.77): calcd.: C, 50.90; H, 3.61; N, 18.26; found: C, 50.73; H, 3.84; N, 18.39.

##### 1-(4-Bromophenyl)-3-(6-methylimidazo[2,1-*b*]thiazol-5-yl)urea (**8f**)

Yield = 50%; m.p. = 288–289 °C; IR: 1709 (>C=O), 3263 (>N-H), 3360 (>N-H); ^1^H NMR: 2.08 (s, 3H, -CH_3_), 7.09 (d, *J* = 4.5 Hz, 1H, Ar-H), 7.37–7.42 (m, 4H, Ar-Hs), 7.55 (d, *J* = 4.5 Hz, 1H, Ar-H), 8.27 (s, 1H, >N-H), 9.20 (s, 1H, >N-H); ^13^C NMR: 13.37 (-CH_3_), 112.09, 113.87, 118.48, 119.00, 120.96 (2C), 131.92 (2C), 137.30, 139.79, 144.87, 154.12 (>C=O); Anal. for:C_13_H_11_BrN_4_OS (351.22): calcd.: C, 44.46; H, 3.16; N, 15.95; found: C, 44.68; H, 3.28; N, 16.17.

##### 4-(3-(6-Methylimidazo[2,1-*b*]thiazol-5-yl)ureido)benzenesulfonamide (**8g**)

Yield = 56%; m.p. = 283–284 °C; IR: 1708 (>C=O), 3120 (>N-H), 3275 (>N-H); ^1^H NMR: 2.09 (s, 3H, -CH_3_), 7.09 (d, *J* = 4.5 Hz, 1H, Ar-H), 7.17 (s, 2H, -NH_2_), 7.58 (d, *J* = 9.0 Hz, 1H, Ar-H), 7.57 (t, *J* = 8.5 Hz, 3H, Ar-Hs), 7.68 (d, *J* = 8.5 Hz, 2H, Ar-Hs), 8.26 (s, 1H, >N-H), 9.35 (s, 1H, >N-H); ^13^C NMR: 13.37 (-CH_3_), 112.18, 118.26, 118.32 (2C), 119.01, 127.21 (2C), 137.39, 137.55, 143.42, 144.98, 153.99 (>C=O); Anal. for C_13_H_13_N_5_O_3_S_2_ (351.40); calcd.: C, 44.43; H, 3.73; N, 19.93; found: C, 44.25; H, 3.56; N, 20.11.

### 3.2. Anticancer Activity

#### 3.2.1. MTT Assay

The developed compounds were evaluated in vitro using the MTT cell viability assay following the published techniques [42,43] (Supplementary Data, page 42).

#### 3.2.2. VEGFR-2 Kinase Activity

This assay was performed using the methodology described below. (Supplementary Data, page 42) [44,45].

#### 3.2.3. PCR Assay

The gene expression levels of apoptosis genes were evaluated in MCF-7 cells using the following approaches in the Supplementary Data, page 43.

#### 3.2.4. Cell Cycle Analysis and Apoptosis

These were performed using the approach described in the Supplementary Data, page 44 [46].

### 3.3. Docking Protocol

Docking studies were performed using AutoDockVina 1.5.7 (Supplementary Data, page 44).

### 3.4. In Silico Predictive ADME Study

The *ADME* study was performed using the SWISSADME server [47] (Supplementary Data, page 45).

## 4. Conclusions

Three derivatives, **6b**, **6i** and **6j**, of isatin–imidazo[2,1-*b*]thiazole hybrids with a carbohydrazide function as a linker showed significant anti-cancerous activity using the MTT assay, with the most active candidates being submitted for further investigation to test their invitro VEGFR-2 inhibitory efficacy compared to Sorafenib as a reference drug. The potent candidate **6i** was then subjected to a PCR study to measure Bax, Bcl2, caspase 8, caspase 9 and cytochrome C to understand the potency of the most active candidate **6i** on malignant cells. Ultimately, for the full investigation regarding its activity, **6i** was utilized for the cell cycle and apoptosis. It additionally arrested the cell cycle in the G2/M phase and induced apoptosis at rates higher than a standard drug. A docking protocol and ADME study were also performed and our candidates had the same binding mode of Sorafenib through interactions with the amino acid residues Lys868, Cys1045, Glu885 and Asp1046. As a consequence, **6i** was specified as a promising lead for additional research to design efficient anti-cancer agents.

## Figures and Tables

**Table 1:** Anti-proliferative activity of **6a**–**l** and **8a**–**g** toward MCF-7 cell line.

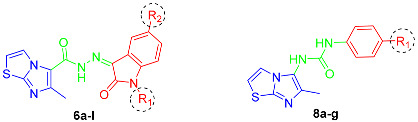			
**Compound**	**R_1_**	**R_2_**	**IC_50_ (µM)**
**6a**	H	H	23.97 ± 0.67
**6b**	H	F	11.50 ± 0.52
**6c**	H	Cl	43.10 ± 3.69
**6d**	H	Br	22.80 ± 1.38
**6e**	*n* -propyl	H	61.39 ± 3.15
**6f**	*n* -propyl	F	82.55 ± 2.75
**6g**	*n* -propyl	Cl	52.47 ± 1.26
**6h**	*n* -propyl	Br	74.34 ± 2.19
**6i**	*n* - butyl	H	8.38 ± 0.62
**6j**	*n* - butyl	F	11.67 ± 0.52
**6k**	*n* - butyl	Cl	42.72 ± 2.01
**6l**	*n* - butyl	Br	52.55 ± 2.36
**8a**	H	–	109.68 ± 4.62
**8b**	CH_3_	–	114.61 ± 4.99
**8c**	OCH_3_	–	106.43 ± 5.32
**8d**	F	–	72.33 ± 4.71
**8e**	Cl	–	35.72 ± 3.32
**8f**	Br	–	31.12 ± 2.47
**8g**	SO_2_NH_2_	–	41.57 ± 1.59
Sorafenib (**I**)	–	–	7.55 ± 0.40

**Table 2:** IC_50_ values of **6b**, **6i** and **6j** toward MCF-10A.

Compound	IC_50_ (µM)
**6b**	125.26 ± 2.7
**6i**	54.63 ± 1.29
**6j**	47.73 ± 0.53
Sorafenib (**I**)	22.35 ± 1.29

**Table 3:** In vitro VEGFR-2 inhibitory activity for **6b**, **6i** and **6j**.

Compounds	R_1_	R_2_	IC_50_ (µM)
**6b**	H	F	3.85 ± 0.15
**6i**	*n* -butyl	H	0.33 ± 0.01
**6j**	*n* -butyl	F	1.51 ± 0.06
Sorafenib (**I**)	–	–	0.09 ± 0.004

**Table 4:** Fold change of apoptosis genes in treated MCF-7 cells with **6i**.

RT-PCR Fold Change					
	Bax	Caspase-8	Caspase-9	Cytochrome C	Bcl-2
**6i**/MCF-7	4.337	2.727	4.947	2.420	0.359
Control MCF-7	1	1	1	1	1

**Table 5:** Cell cycle of compound **6i** in MCF-7.

	%G0–G1	%S	%G2/M
**6i**/MCF-7	53.66	19.27	27.07
Cont. MCF-7	64.73	23.96	11.31

**Table 6:** Apoptosis analysis for **6i** in MCF-7.

	Total	Early	Late	Necrosis
**6i**/MCF-7	32.81	22.05	7.61	3.15
Cont. MCF-7	1.91	0.43	0.11	1.37

## Data Availability

Data is contained within the article and supplementary material.

## Supplementary Materials

The following supporting information can be downloaded at: https://www.mdpi.com/article/10.3390/ph17020216/s1, Figures S1 and S2 and Table S1 (Molecular docking); Figure S3 and Table S2 (Virtual ADME assessment); Figures S4–S19 (^1^H NMR and ^13^C NMR spectra of **6a**–**l**); Figures S20–S33 (^1^H NMR and ^13^C NMR spectra of **8a**–**g**).
