# Supplementary material for: Design, Synthesis and Biological Assessment of N′-(2-Oxoindolin-3-ylidene)-6-methylimidazo[2,1-b]thiazole-5-carbohydrazides as Potential Anti-Proliferative Agents toward MCF-7 Breast Cancer
Source: Pharmaceuticals (Basel). 2024 Feb 7;17(2):216. doi: 10.3390/ph17020216 (PMC10892120; doi:10.3390/ph17020216)
Supplement: Supplementary file 1 [file pharmaceuticals-17-00216-s001.zip › pharmaceuticals-2849772-supplementary.pdf]

Supplementary data

Design, Synthesis and Biological Assessment of N0-(2-Oxoindolin-3-ylidene)-6-methylimidazo[2,1-b]thiazole-5-carbohydrazides as Potential Anti-Proliferative Agents toward MCF-7 Breast Cancer

Najla A. Alshaye [1], Mohamed K. Elgohary [2,*], Mahmoud S. Elkotamy [2] and Hatem A. Abdel-Aziz [3,*]

[1] Department of Chemistry, College of Science, Princess Nourah bint Abdulrahman University, P.O. Box 84428, Riyadh 11671, Saudi Arabia; naalshaye@pnu.edu.sa
[2] Pharmaceutical Chemistry Department, Faculty of Pharmacy, Egyptian-Russian University, Cairo 11829, Egypt; mahmoud-elkotamy@eru.edu.eg
[3] Applied Organic Chemistry Department, National Research Center, Dokki, Cairo 12622, Egypt
* Correspondence: mohamed-elgohary@eru.edu.eg (M.K.E.); ha.abdel-aziz@nrc.sci.eg (H.A.A.-A.)

Contents

Molecular docking	4
**Figure S1**. Alignment of the original ligand sorafenib (pink) and re-docked ligand (blue)……..	5
**Table S1**. Docking and interaction pattern of most active compounds **6b**, **6i** and **6j** on VEFGR-2 active site………………..………………………………………………….	5
**Figure S2**. 3D diagram of targeted compounds binds with VEGFR binding site ………..	6
Virtual ADME assessment	7
**Figure S3**. Computational ADME prediction study of human intestinal absorption %, BBB Permeation and p-glycoprotein permeability of all designed compounds…………………...	8
**Table S2**. ADME and predicted pharmacological aspects for designed compounds **6a-l** and **8a-g**……………………………………………………………………………….	9
^{1}H NMR and ^{13}C NMR spectra	10
**Figure S4**. ^{1}H NMR of compound **6a**…………………………………………………..…	11
**Figure S5**. ^{13}C NMR of compound **6a**…………………………………………………	12
**Figure S6**. ^{1}HNMR of compound **6b**………………………………………………..……	13
**Figure S7**. ^{13}CNMR of compound **6b**…………………………………………………	14
**Figure S8**. ^{1}HNMR of compound **6c**………………………………………………..……	15
**Figure S9**. ^{13}CNMR of compound **6c**…………………………………………...……	16
**Figure S10**. ^{1}HNMR of compound **6d**………………………………………………………	17
**Figure S11**. ^{13}CNMR of compound **6d**……………………………………..……..……	18
**Figure S12**. ^{1}HNMR of compound **6h**………………………………………………………	19
**Figure S13**. ^{13}CNMR of compound **6h**………………………………………………	20
**Figure S14**. ^{13}CNMR of compound **6i**………………………………………………	21
**Figure S15**. ^{13}CNMR of compound **6i**………………………………………………	22
**Figure S16**. ^{13}CNMR of compound **6j**………………………………………………	23
**Figure S17**. ^{13}CNMR of compound **6j**………………………………………………	24
**Figure S18**. ^{1}HNMR of compound **6l**…………………………………….…………………	25
**Figure S19**. ^{13}CNMR of compound **6l**………………………………….………………	26
**Figure S20**. ^{1}HNMR of compound **8a**……………………………….………………	27
**Figure S21**. ^{13}CNMR of compound **8a**………………………………………………	28

Figure S22. ^{1}HNMR of compound **8b**.. **29**
Figure S23. ^{13}CNMR of compound **8b**.. **30**
Figure S24. ^{1}HNMR of compound **8c**.. **31**
Figure S25. ^{13}CNMR of compound **8c**.. **32**
Figure S26. ^{1}HNMR of compound **8d**.. **33**
Figure S27. ^{13}CNMR of compound **8d**.. **34**
Figure S28. ^{1}HNMR of compound **8e**.. **35**
Figure S29. ^{13}CNMR of compound **8e**.. **36**
Figure S30. ^{1}HNMR of compound **8f**.. **37**
Figure S31. ^{13}CNMR of compound **8f**.. **38**
Figure S32. ^{1}HNMR of compound **8g**.. **39**
Figure S33. ^{13}CNMR of compound **8g**.. **40**
Experimental procedures **41**
Chemistry Instruments.. **42**
MTT assay.. **42**
VEGFR-2 kinase activity **42**
PCR assesment **43**
Cell cycle analysis.. **44**
Docking protocol.. **44**
In silico predictive ADME study.. **45**

Molecular docking

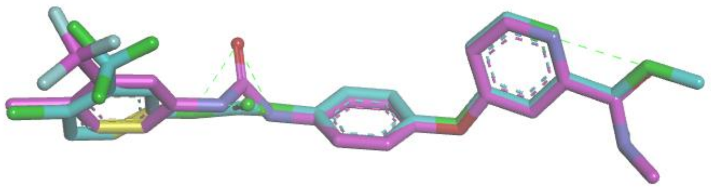

```
mode |   affinity | dist from best mode
     | (kcal/mol) | rmsd l.b.| rmsd u.b.
-----+------------+----------+----------
   1        -11.0      0.000      0.000
   2         -8.8      4.943      6.434
   3         -8.5      3.875      5.950
   4         -8.2      4.874      7.274
Writing output ... done.
```

Figure S1: Alignment of the original ligand sorafenib (pink) and re-docked ligand (blue).

Table S1. docking and interaction pattern of most active compounds **6b, 6i** and **6j** on VEFGR-2 active site

Compound	Binding Score (kcal/mol)	Interaction	Bond length (A°)	Ligand atom	residue	Enzyme	PDB ID
**sorafenib**	-11.0	H-bond	----	----------------	Cys919	VEGFR-2	4ASD
		Halogen	----	----------------	Glu885		
		bond	----	----------------	Asp1046		
		H-bond	----	----------------	Leu1035		
		π-sigma			Cys1045		
		π-sulfur					
**6b**	-8.2	H-bond	2.89	N hydrazide	Asp1046	VEGFR-2	4ASD
		H-bond	3.10	N hydrazide	Glu885		
		π- sigma	3.99	isatin	Leu889		
		π-cation	3.74	imidazothiazole	Lys868		
		π-sigma	4.44	imidazothiazole	Val916		
**6i**	-8.8	H-bond	2.88	N hydrazide	Asp1046	VEGFR-2	4ASD
		π-sigma	3.54	isatin	Leu889		
		π-cation	4.50	imidazothiazole	Lys868		
		π-sigma	3.3	imidazothiazole	Val916		
		π-sulfur	4.84	imidazothiazole	Cys1045		
**6j**	-8.2	H-bond	3.13	N hydrazide	Asp1046	VEGFR-2	4ASD
		H-bond	3.13	C=O of isatin	Lys868		
		π-sulfur	4.71	imidazothiazole	Cys1045		

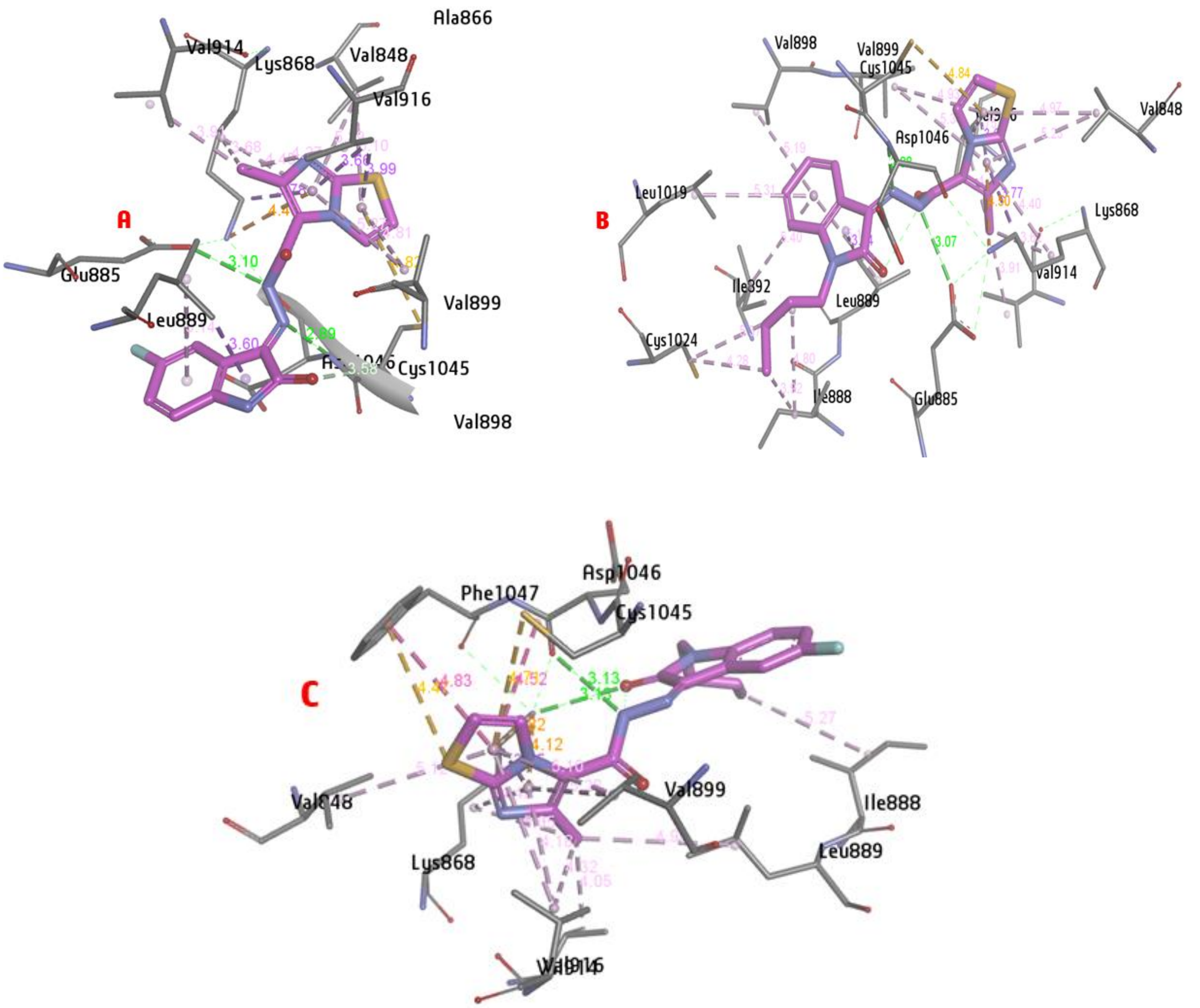

Figure S2. 3D diagram of targeted compounds bind with VEGFR binding site (PDB ID: **4ASD**); **A)** Compound **6b** in ATP binding site of VEGR-2 showing one hydrogen interactions with Asp1046 residue and pi-cation interaction with Lys868; **B)** compound **6i** exhibited essential hydrogen bond interaction with Asp1046 residue, pi-sigma interaction with Cys1045 and pi-cation interaction with Lys868; **C)** Docking pose of compound **6j** demonstrate the same key interactions with VEGFR-2 binding site; hydrogen interactions with Asp1046 and Lys868 residue.

Virtual ADME assessment

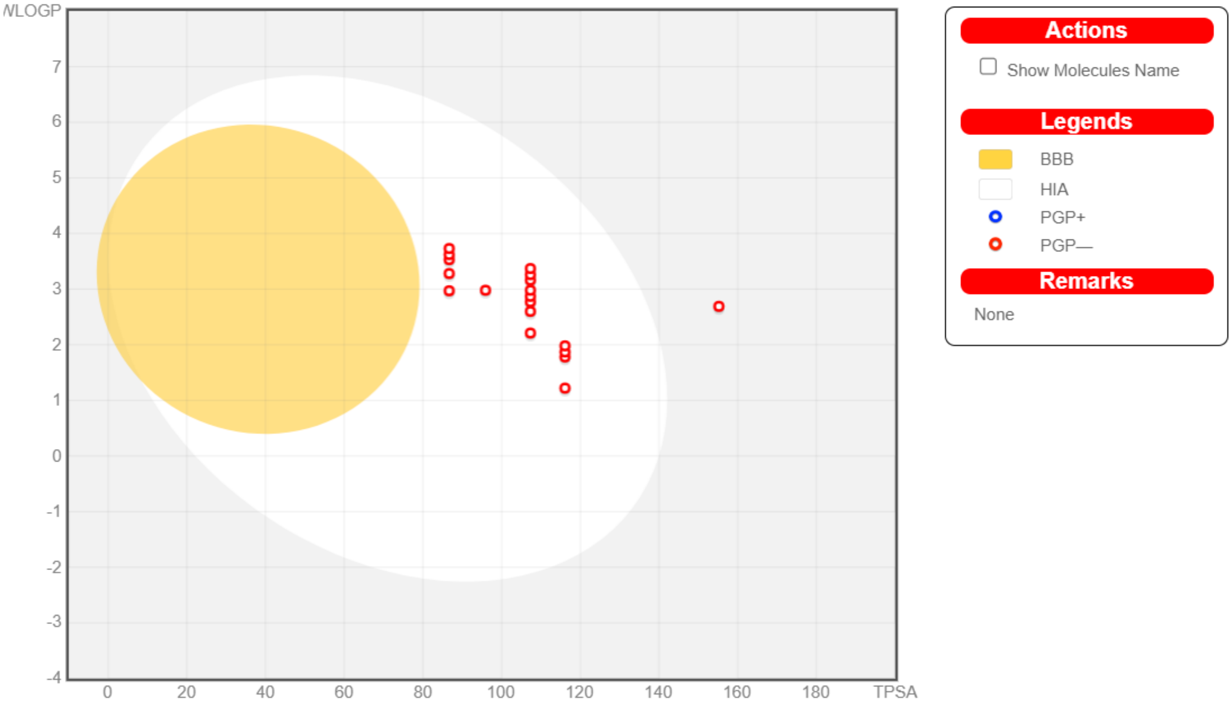

Figure S3. Computational ADME prediction study of human intestinal absorption %, BBB Permeation and p-glycoprotein permeability of all designed compounds

Table S2. ADME and predicted pharmacological aspects for designed compounds **6a-l** and **8a-g**

**Compounds**	**M.wt**	**H. Bond Donor**	**H. Bond Acceptor**	**Log p**	**TPSA**	**Rotatable bonds**	**Lipinski violations**	**Veber violations**	**Pgp substrate**
**6a**	325.35	2	4	1.4	116.1	3	0	0	NO
**6b**	343.34	2	5	1.95	116.1	3	0	0	NO
**6c**	359.79	2	4	2.11	116.1	3	0	0	NO
**6d**	404.24	2	4	2.27	116.1	3	0	0	NO
**6e**	367.42	1	4	2.21	107.31	5	0	0	NO
**6f**	385.42	1	5	2.1	107.31	5	0	0	NO
**6g**	401.87	1	4	2.57	107.31	5	0	0	NO
**6h**	446.32	1	4	2.89	107.31	5	0	0	NO
**6i**	381.45	1	4	2.82	107.31	6	0	0	NO
**6j**	399.44	1	5	2.83	107.31	6	0	0	NO
**6k**	415.9	1	4	2.92	107.31	6	0	0	NO
**6l**	460.35	1	4	2.87	107.31	6	0	0	NO
**8a**	272.33	2	2	2.4	86.67	4	0	0	NO
**8b**	302.35	2	3	2.18	95.9	5	0	0	NO
**8c**	286.35	2	2	2.48	86.67	4	0	0	NO
**8d**	290.32	2	3	2.34	86.67	4	0	0	NO
**8e**	306.77	2	2	2.53	86.67	4	0	0	NO
**8f**	351.22	2	2	2.64	86.67	4	0	0	NO
**8g**	351.4	3	5	1.16	155.21	5	0	1	NO

M.wt: molecular weight.
LogP: Octanol-water partition coefficient
TPSA: Topological polar surface

^{1}H NMR and ^{13}C NMR spectra

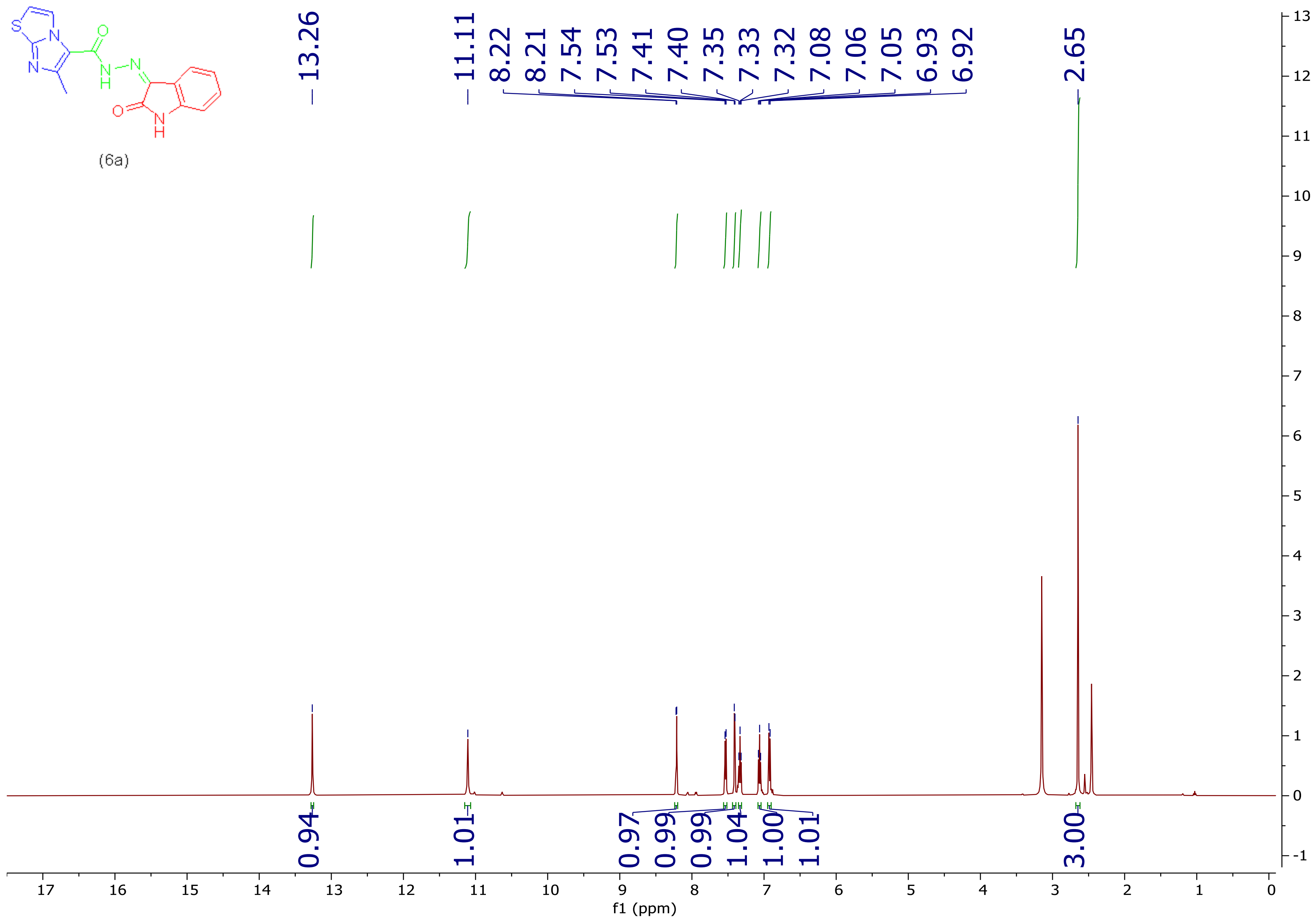

Figure S4. ^{1}HNMR of compound **6a**

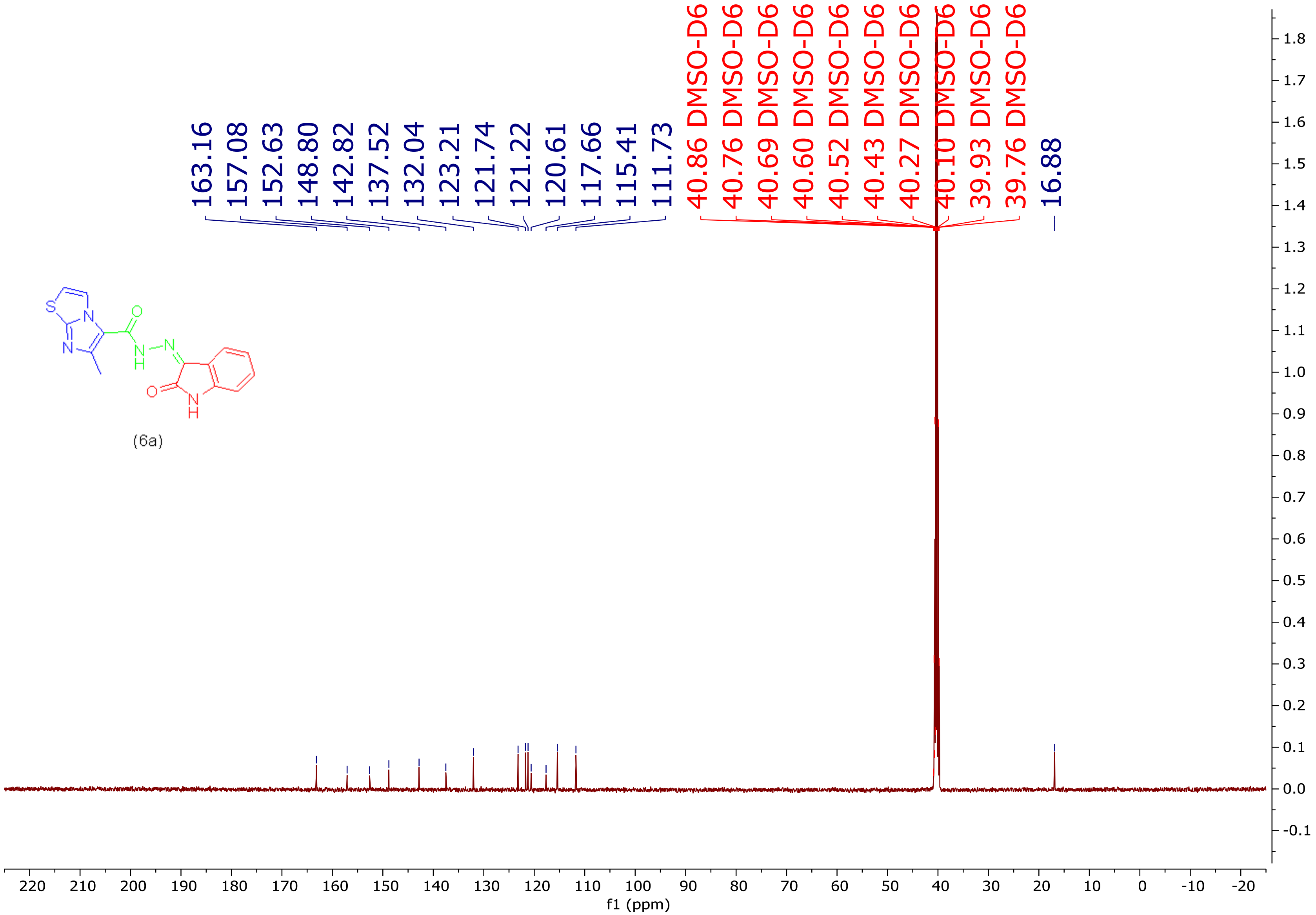

Figure S5. ^{13}C NMR of compound **6a**

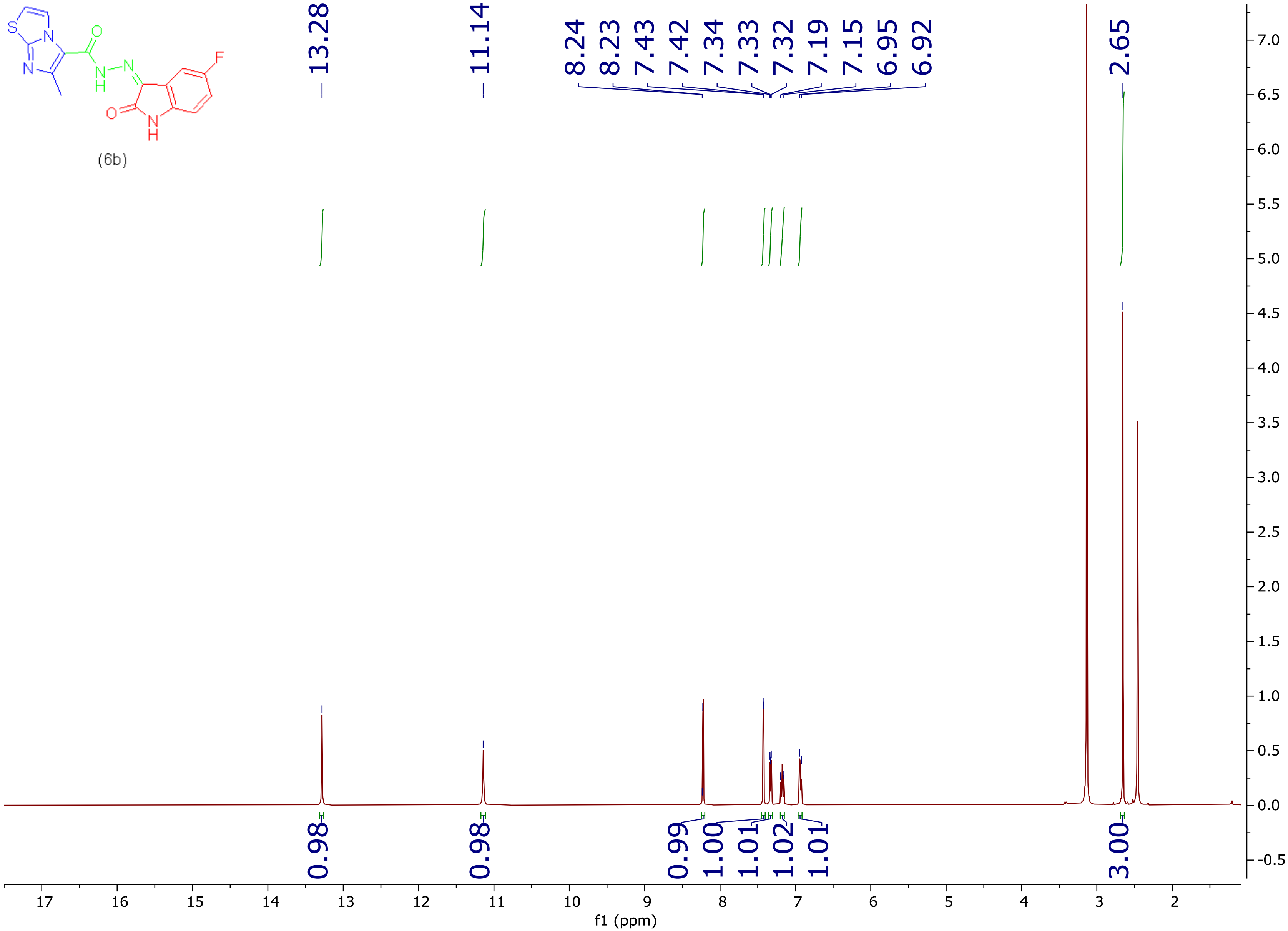

Figure S6. ^{1}HNMR of compound **6b**

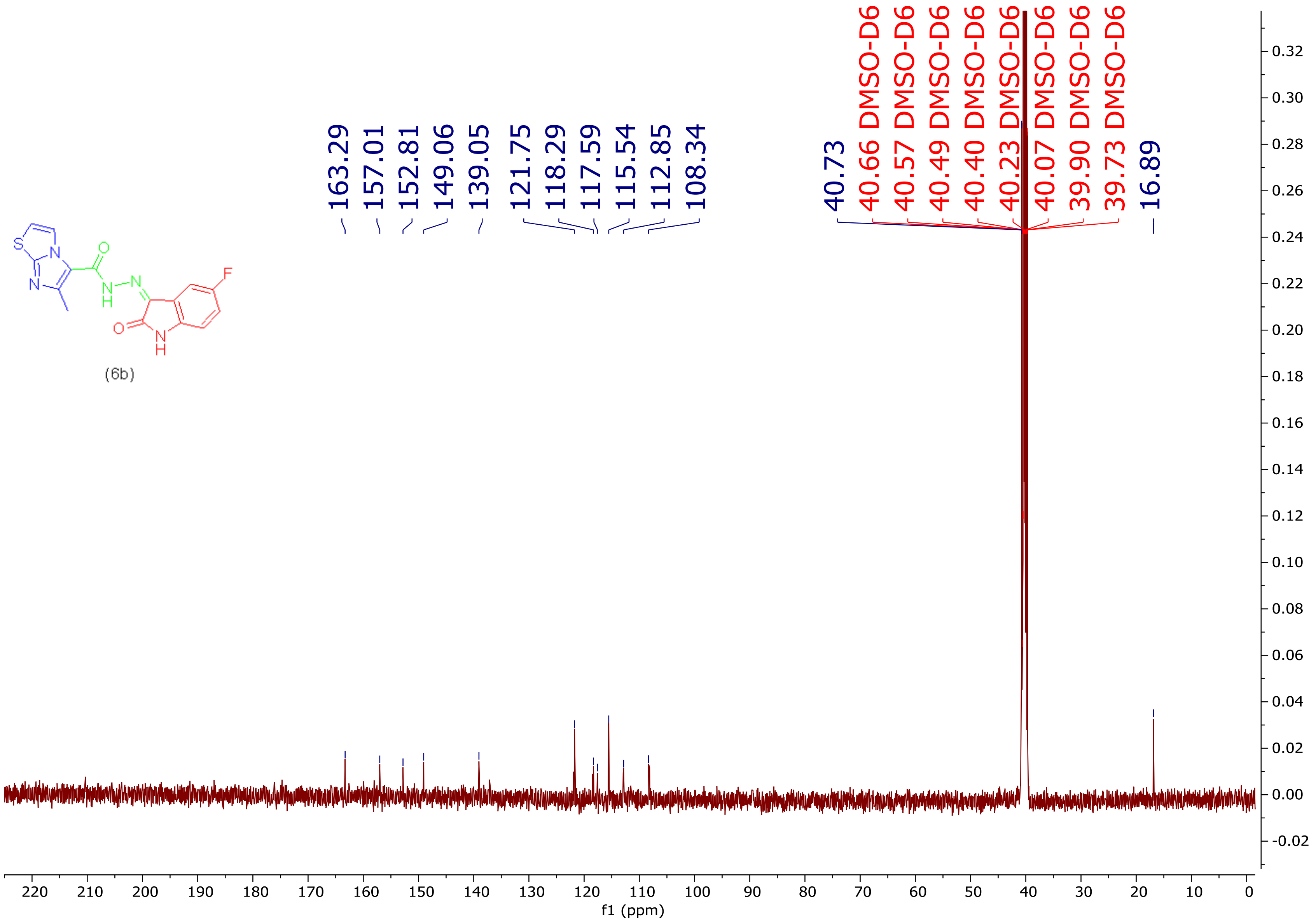

Figure S7. ^{13}C NMR of compound **6b**

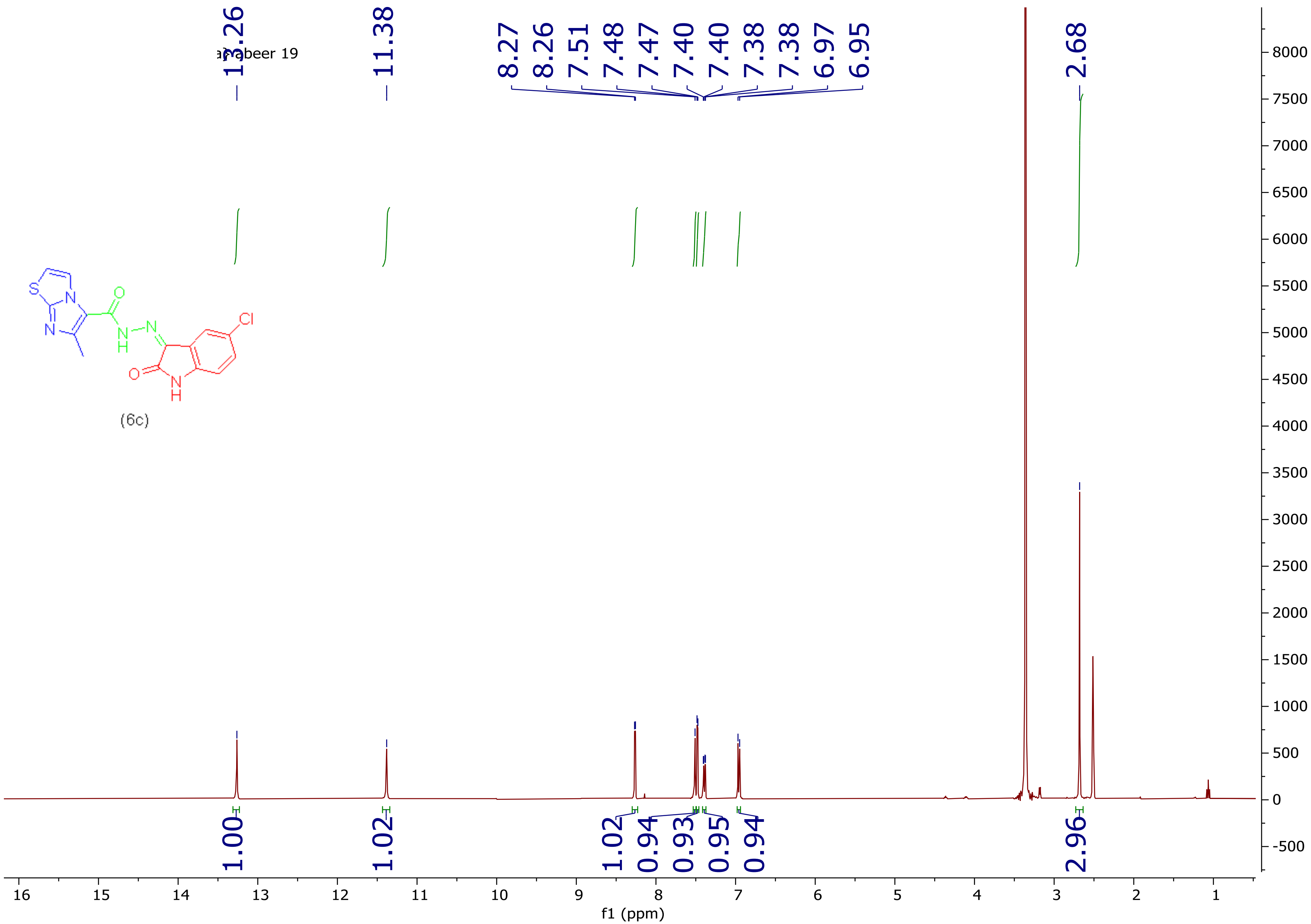

Figure S8. ^{1}HNMR of compound **6c**

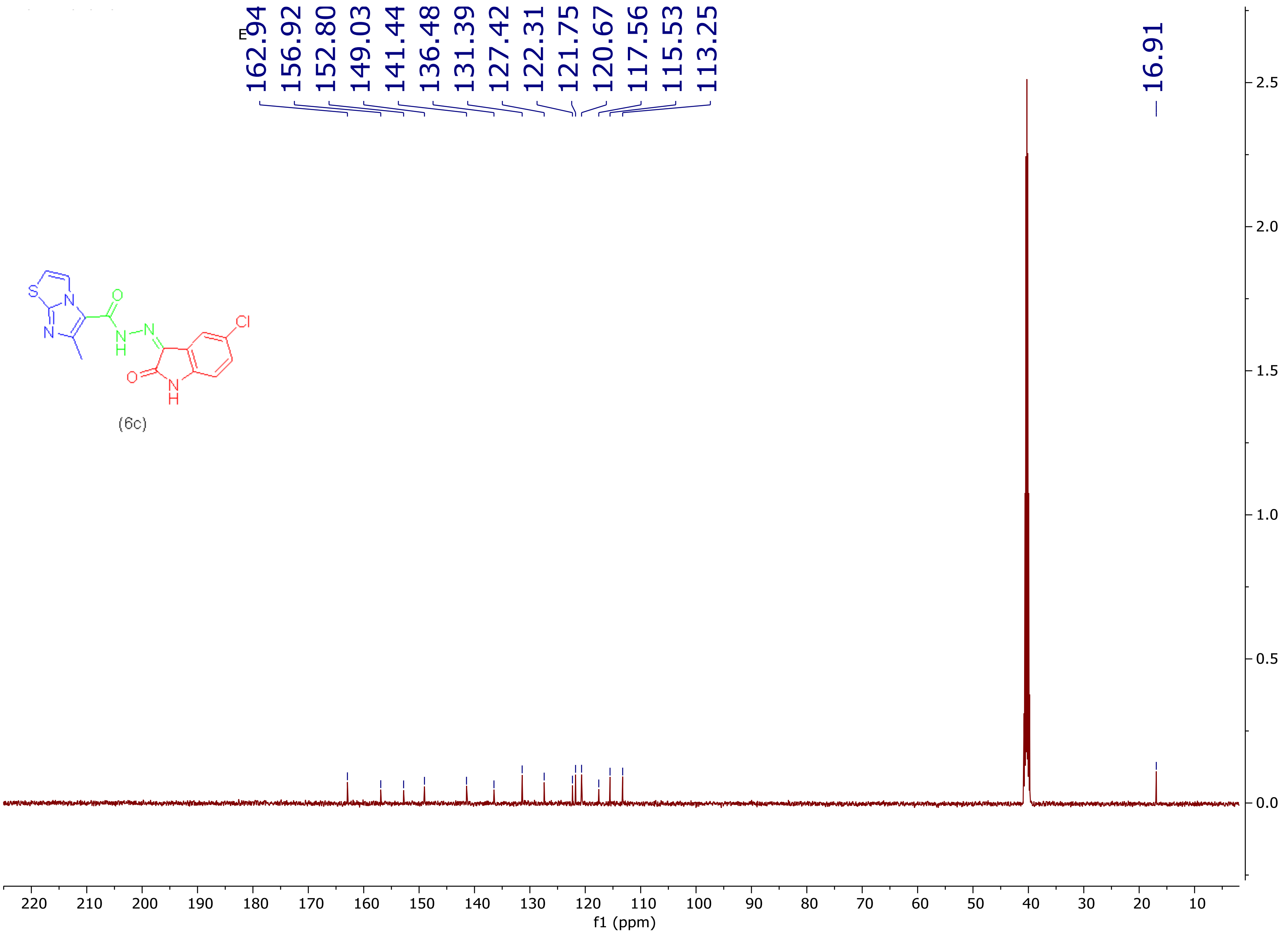

Figure S9. ^{13}C NMR of compound **6c**

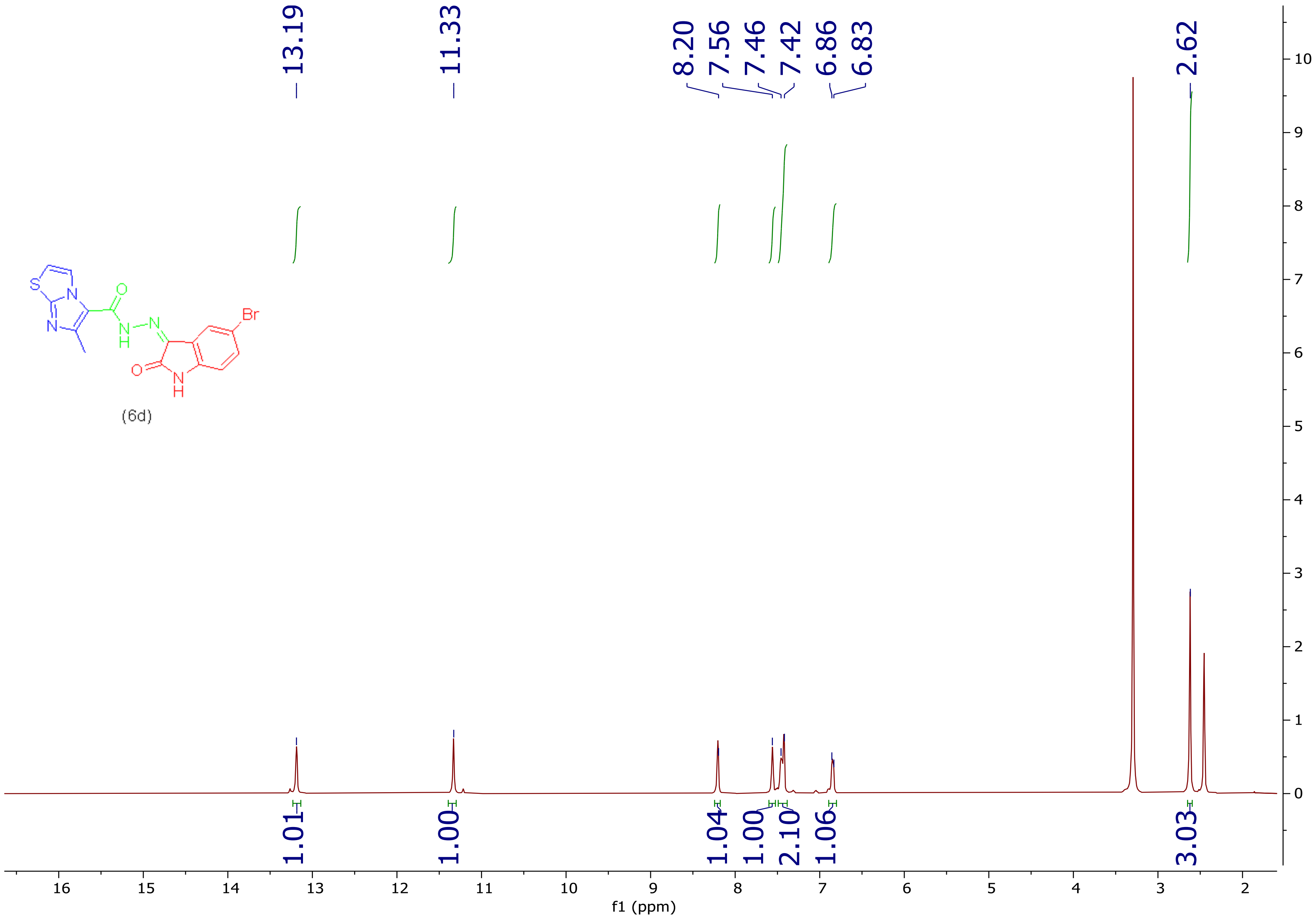

Figure S10. ^{1}HNMR of compound **6d**

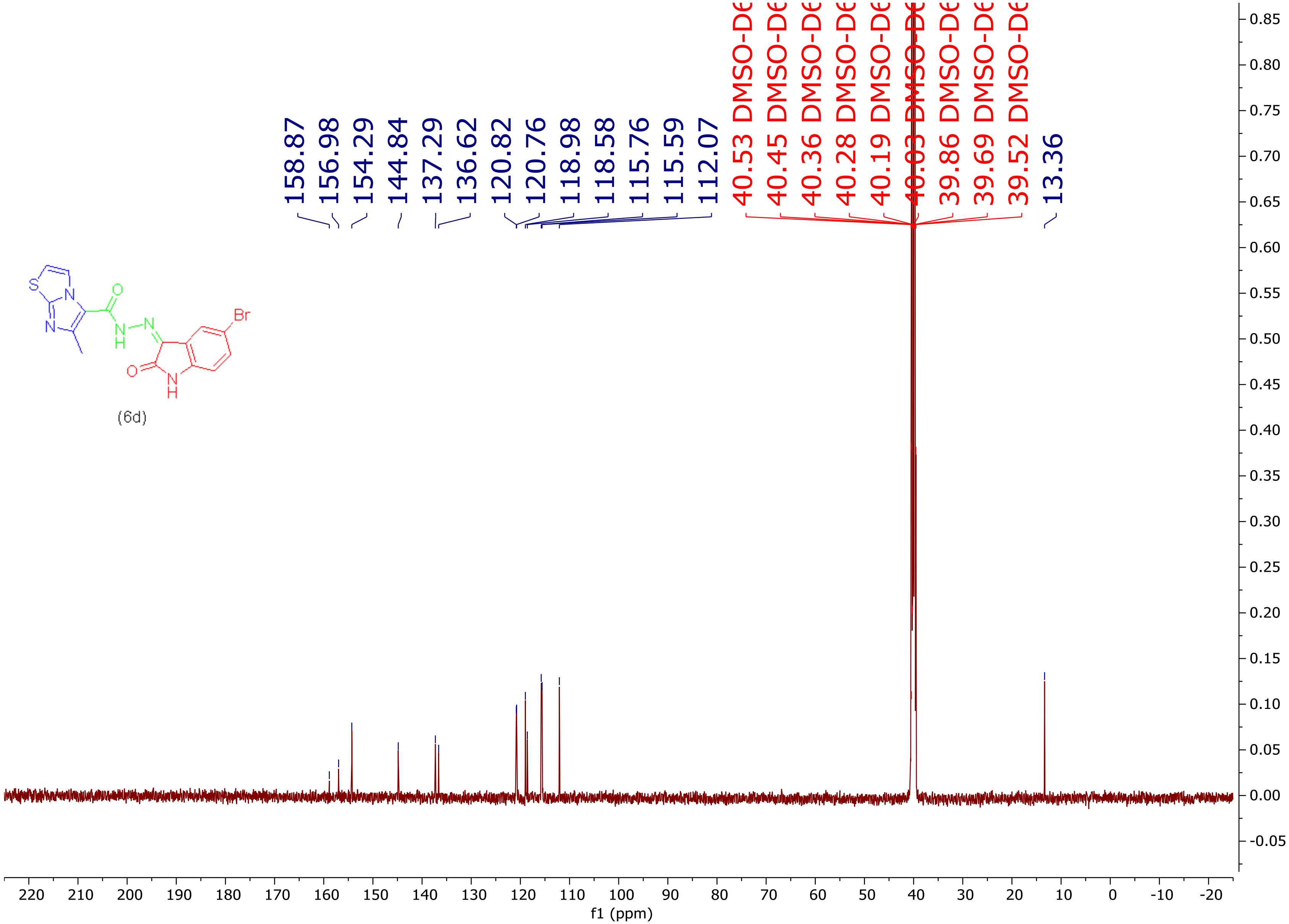

Figure S11. ^{13}C NMR of compound **6d**

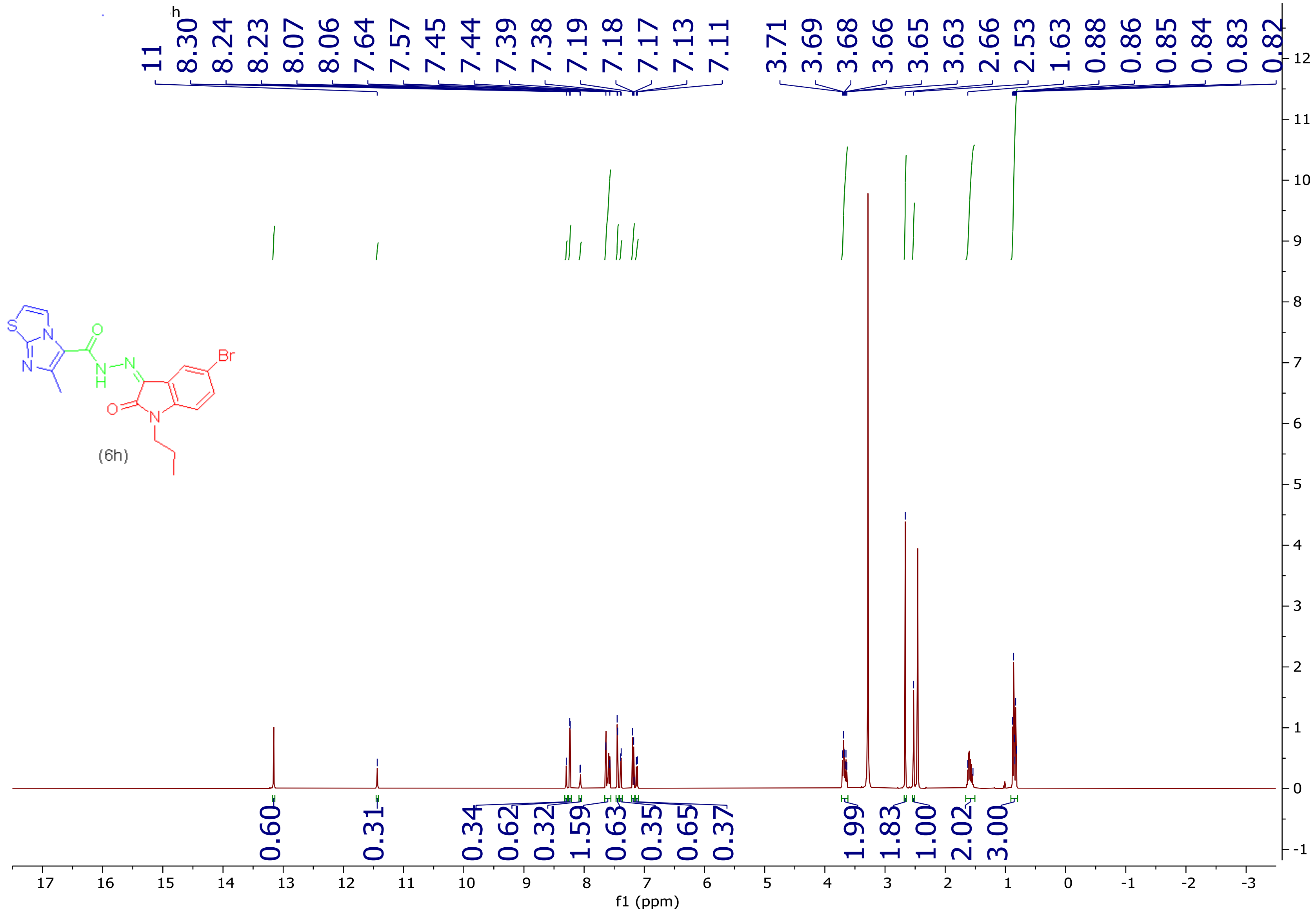

Figure S12. ^{1}HNMR of compound **6h**

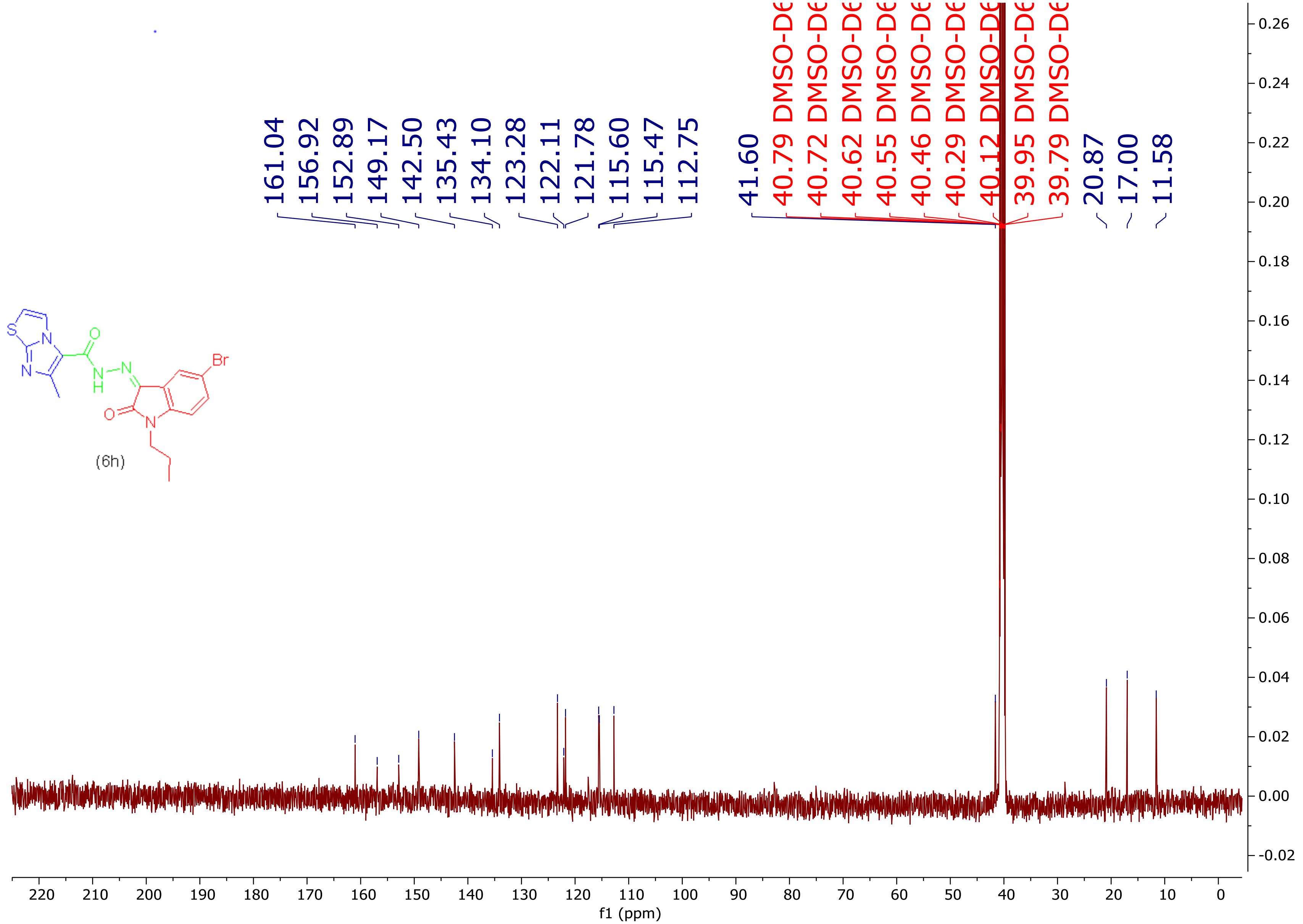

Figure S13. ^{13}C NMR of compound **6h**

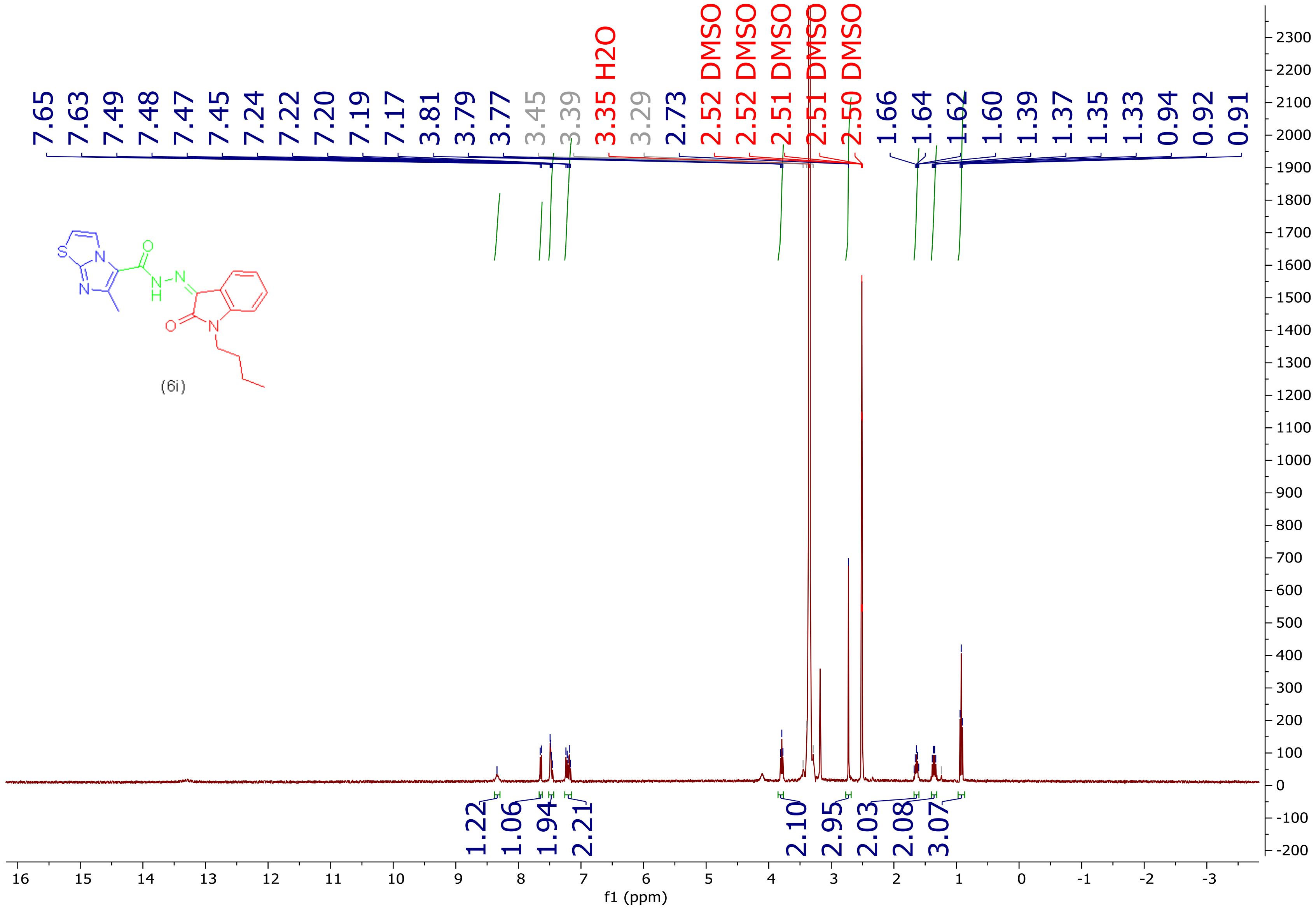

Figure S14. ^{1}HNMR of compound

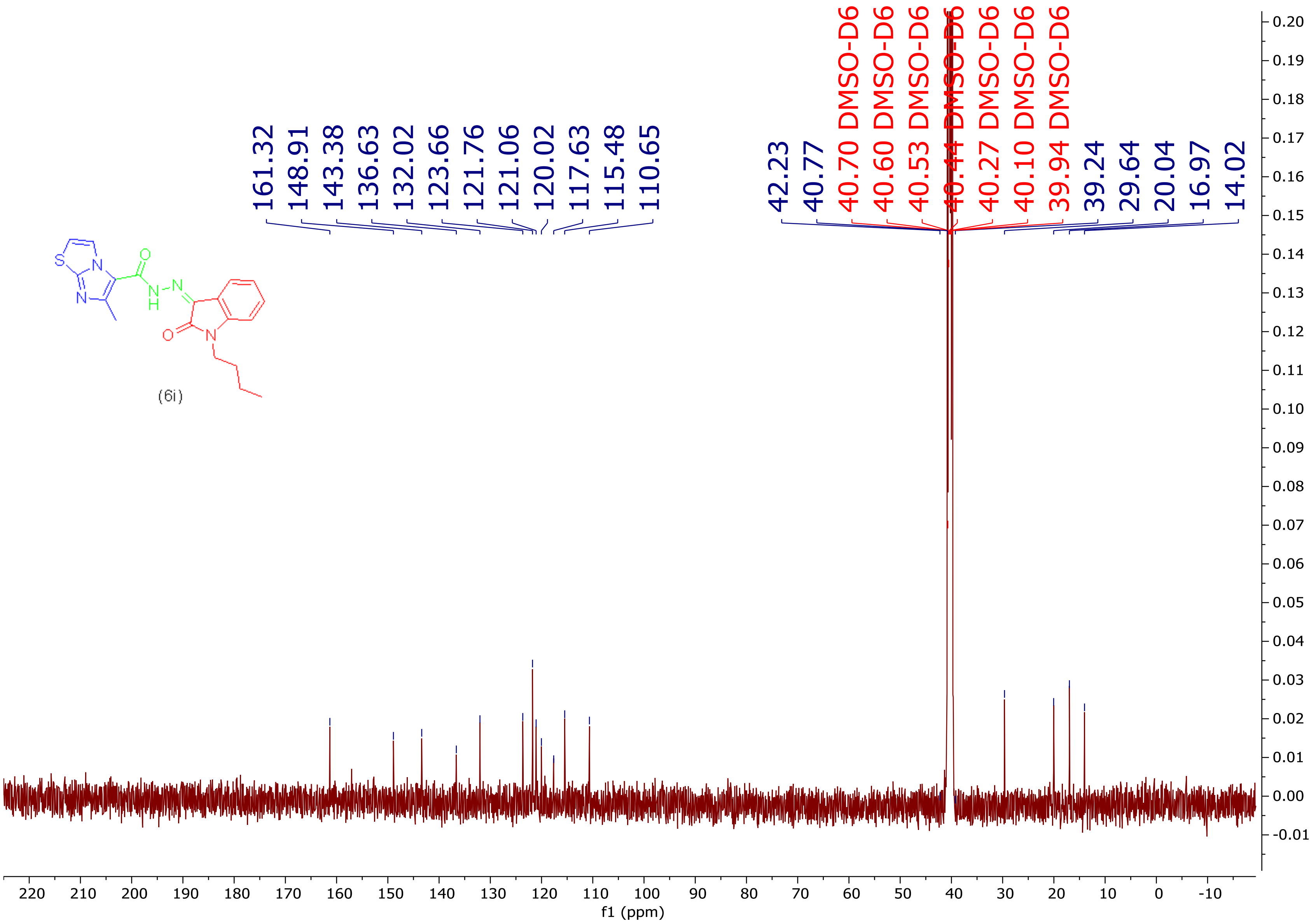

Figure S15. ^{13}C NMR of compound

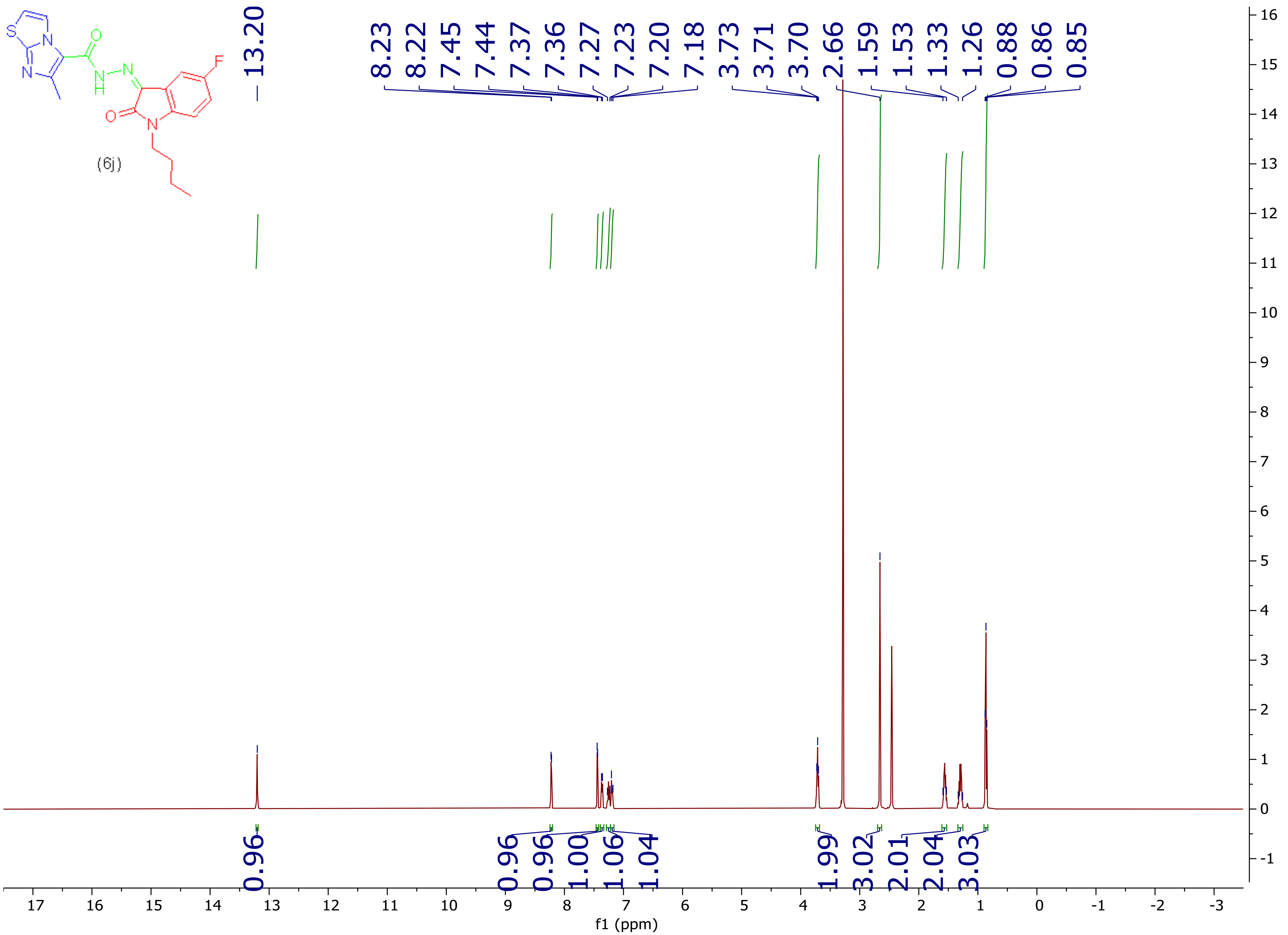

Figure S16. ¹HNMR of compound

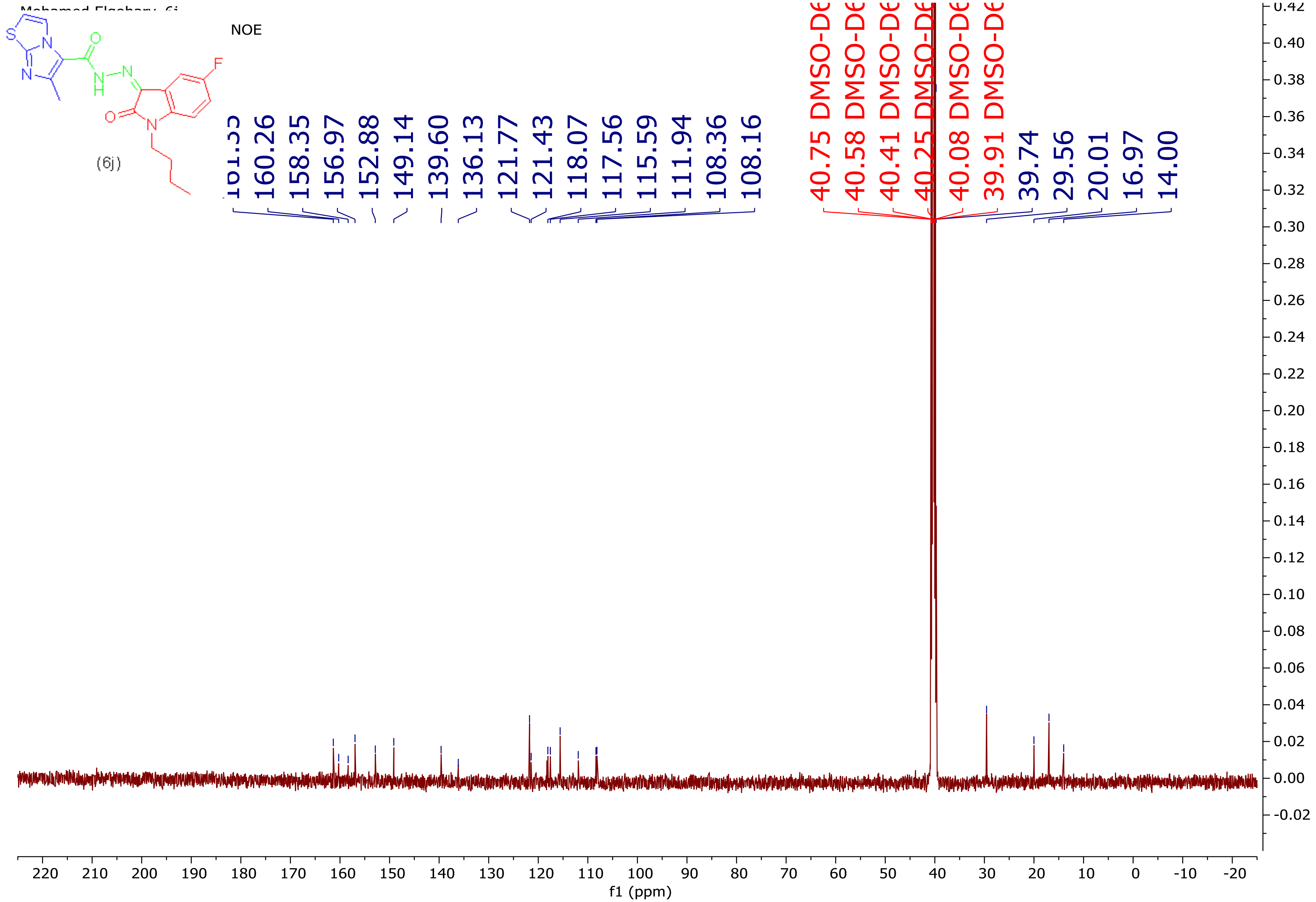

Figure S17. ^{13}C NMR of compound

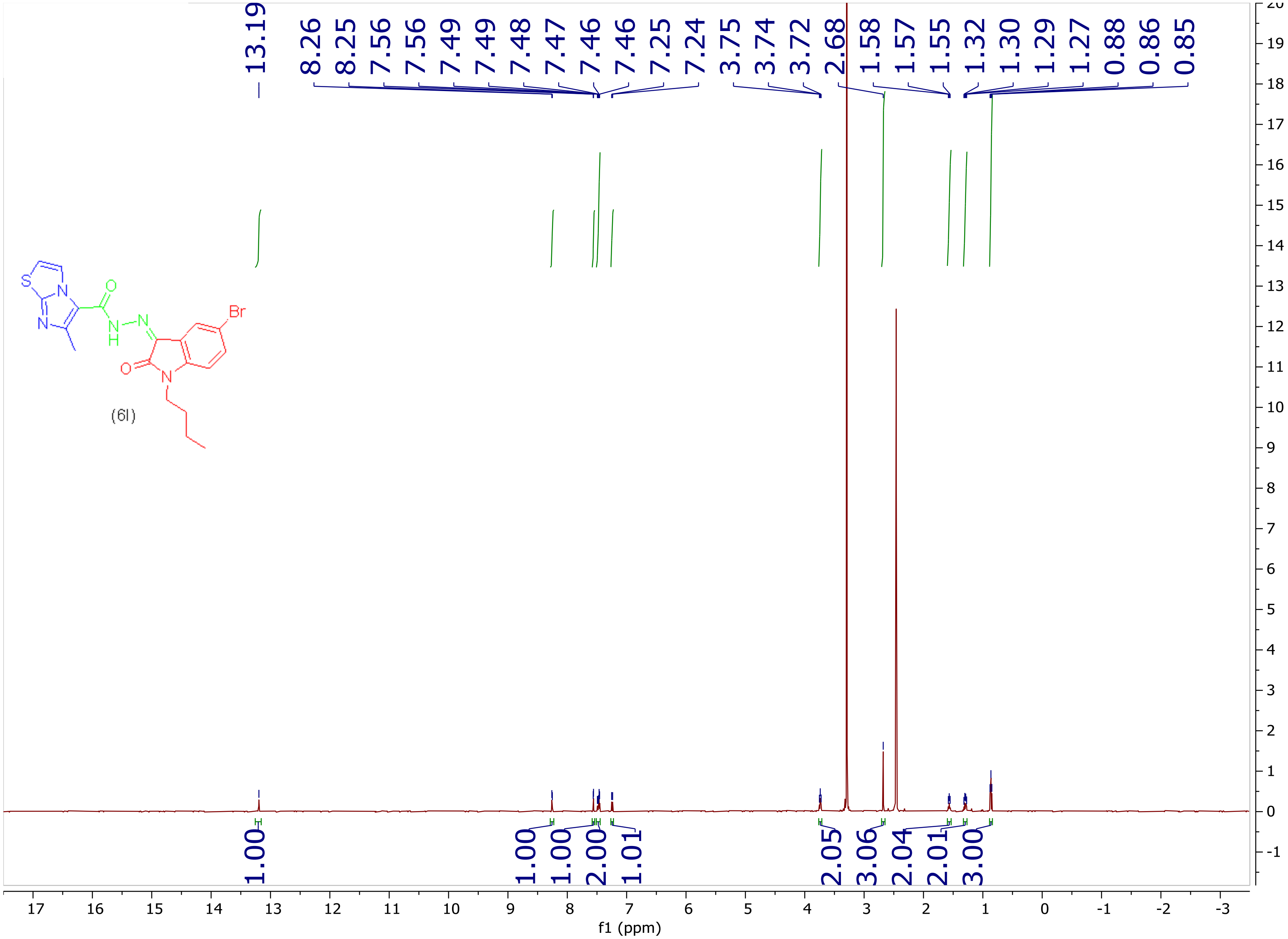

Figure S18. ^{1}HNMR of compound **6l**

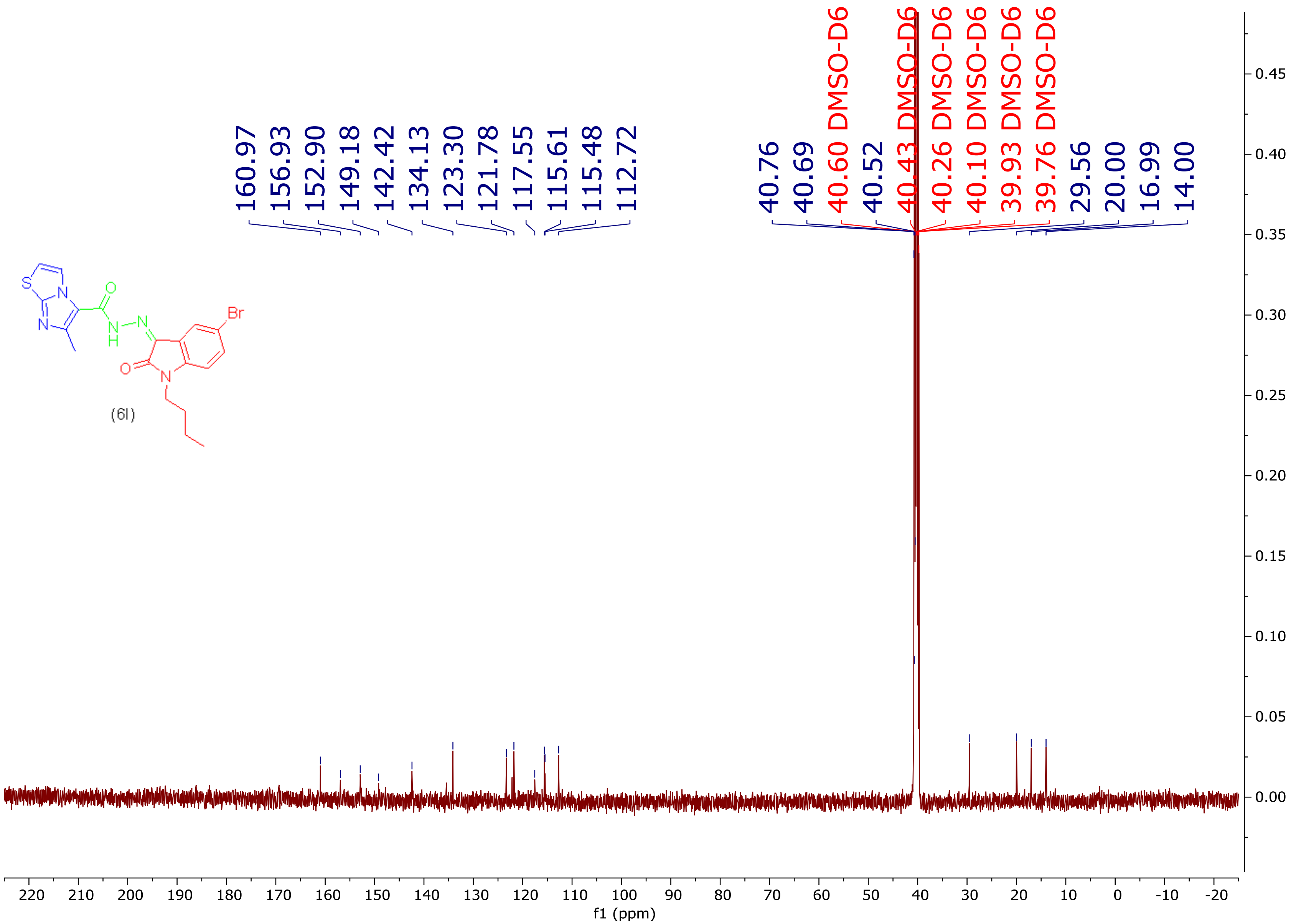

Figure S19. ^{13}C NMR of compound **6l**

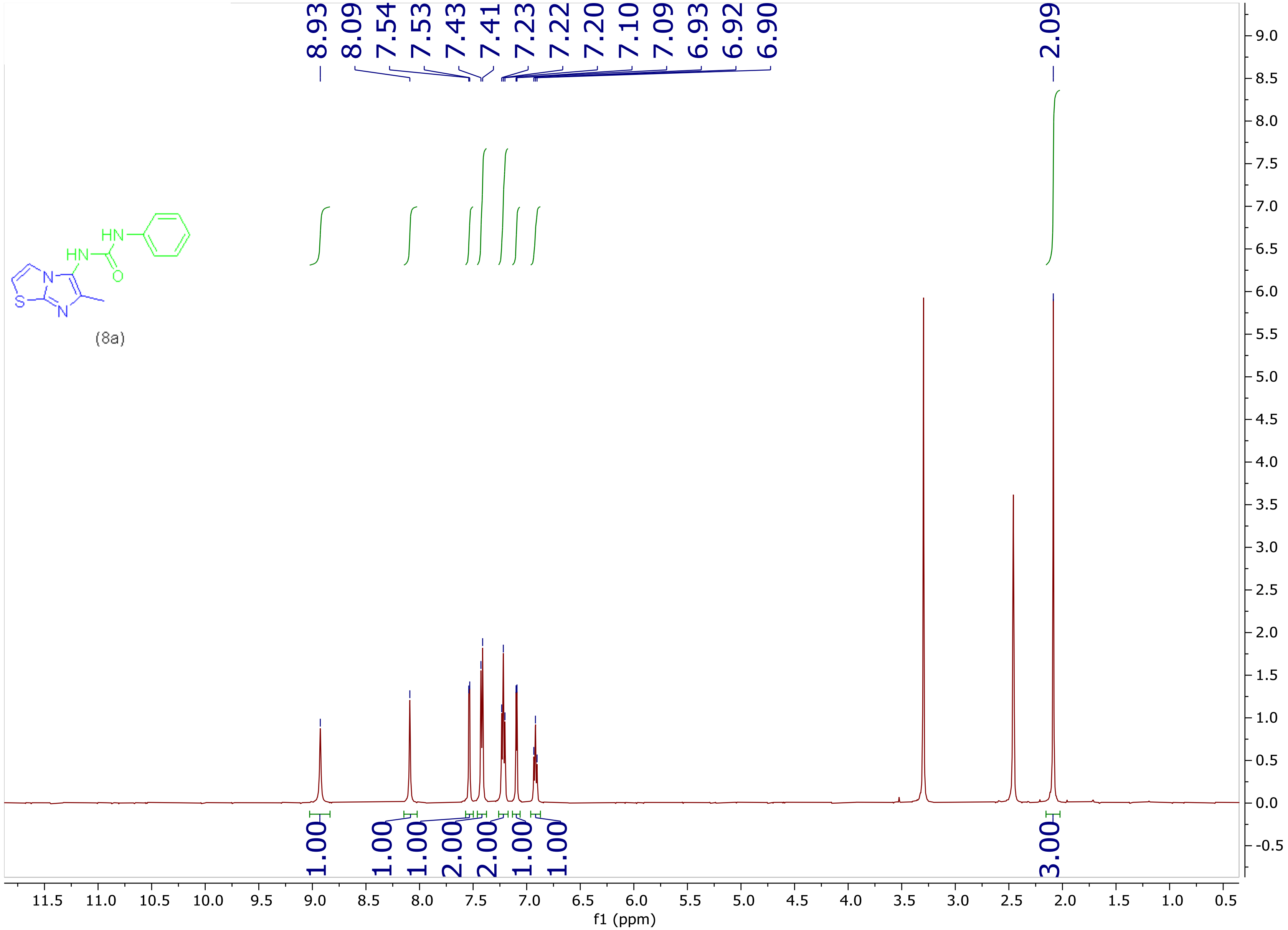

Figure S20. ¹HNMR of compound **8a**

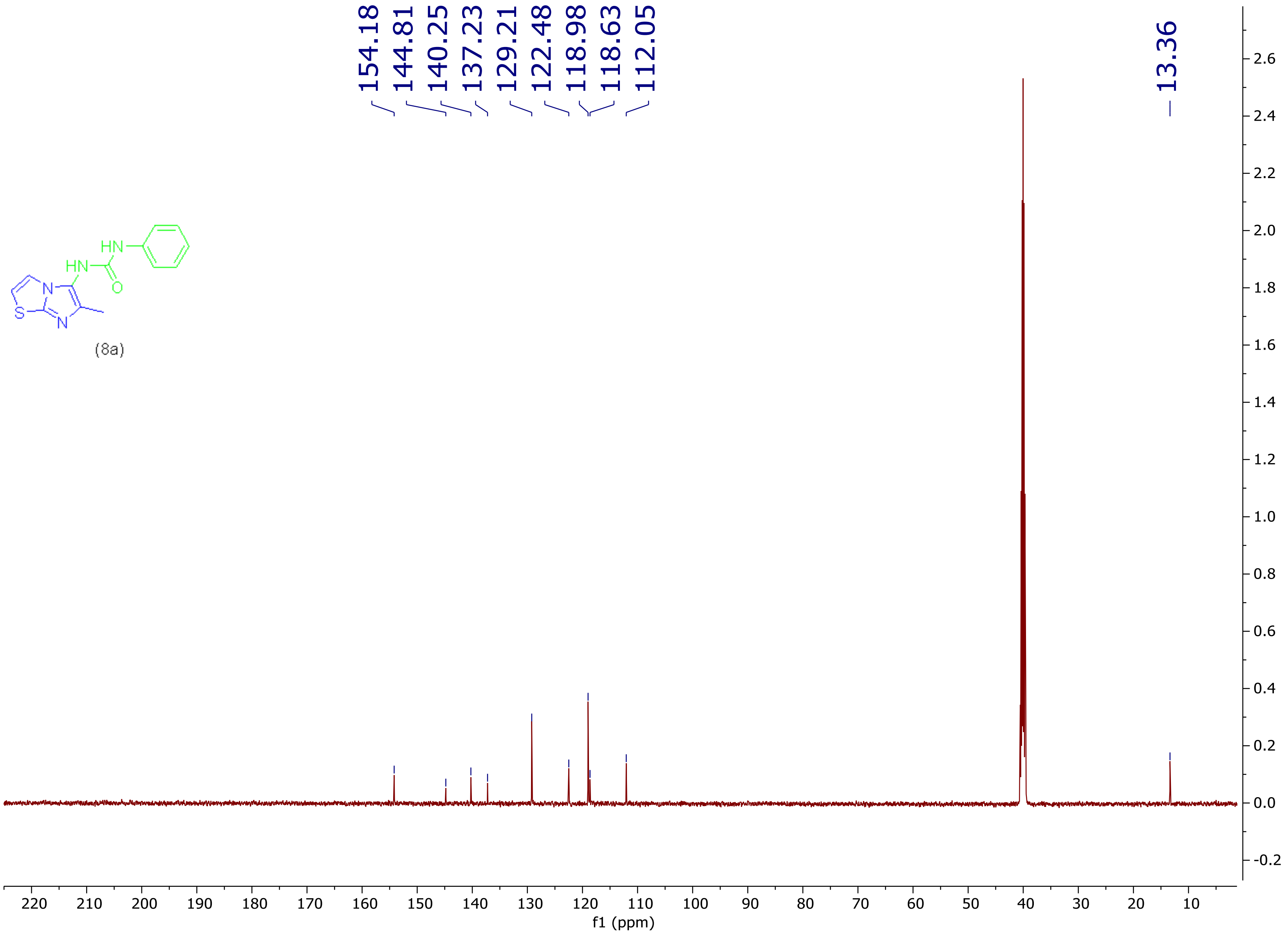

Figure S21. ^{13}C NMR of compound **8a**

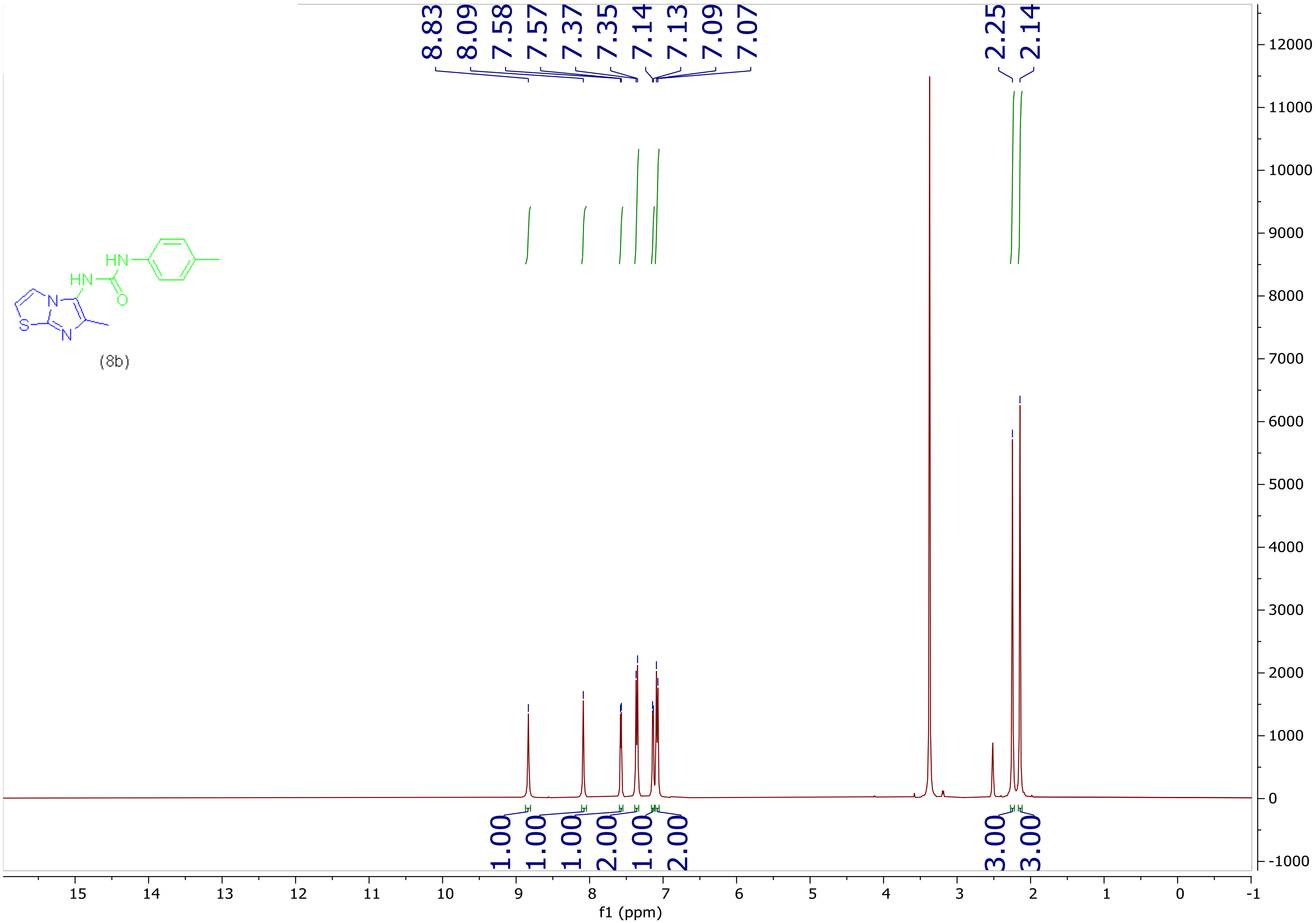

Figure S22. ^{1}H NMR of compound **8b**

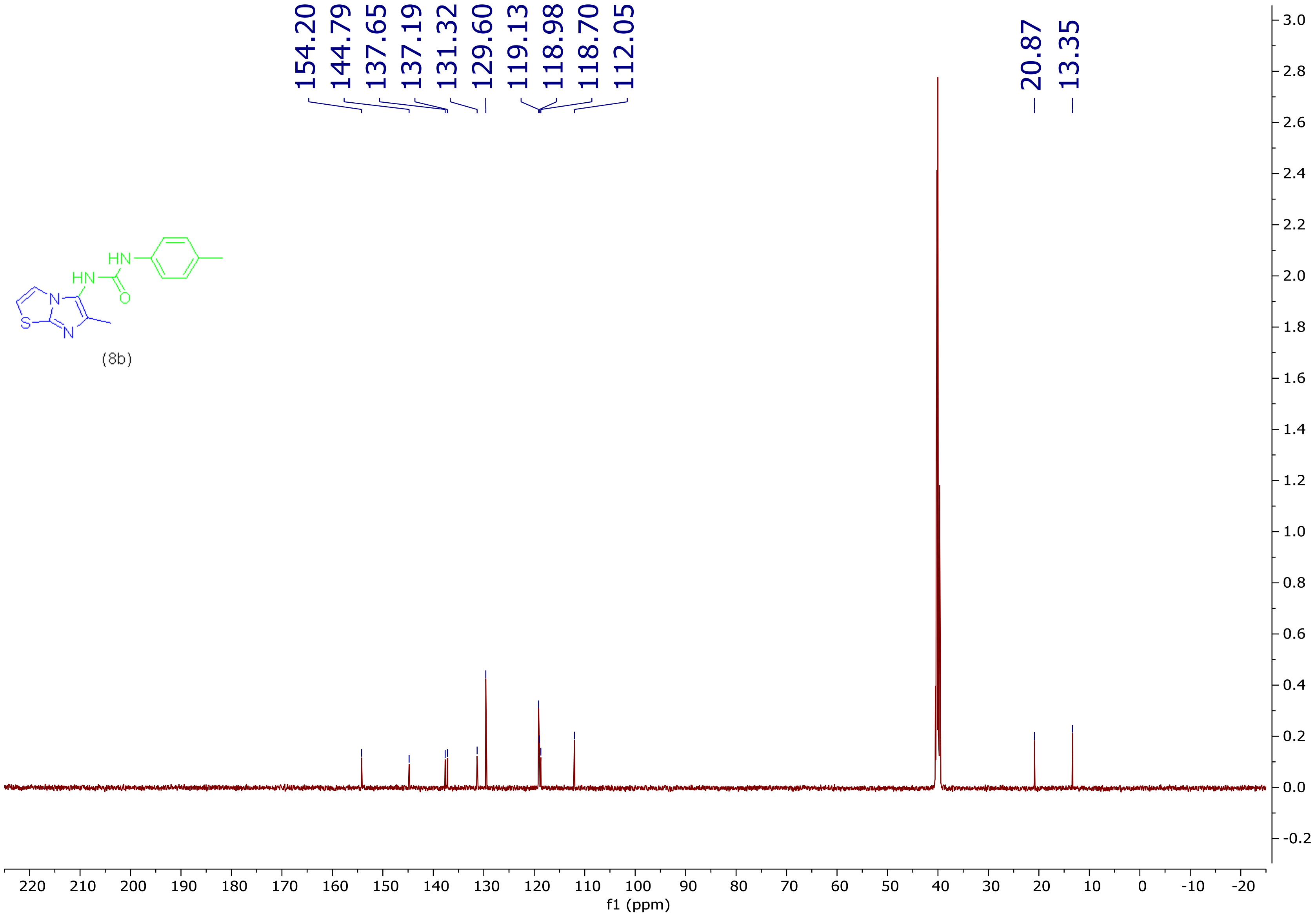

Figure S23. ^{13}C NMR of compound **8b**

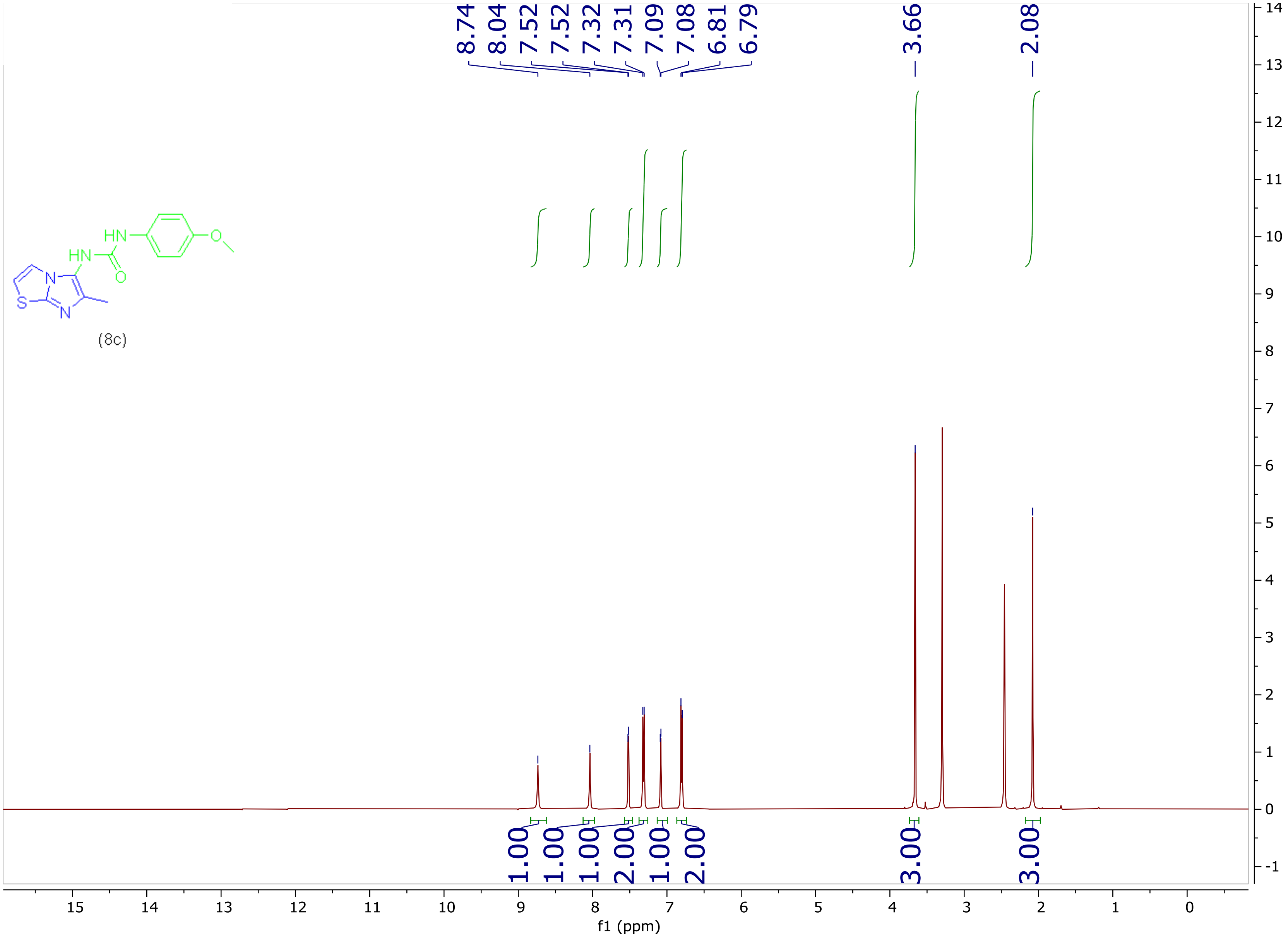

Figure S24. ^{1}H NMR of compound **8c**

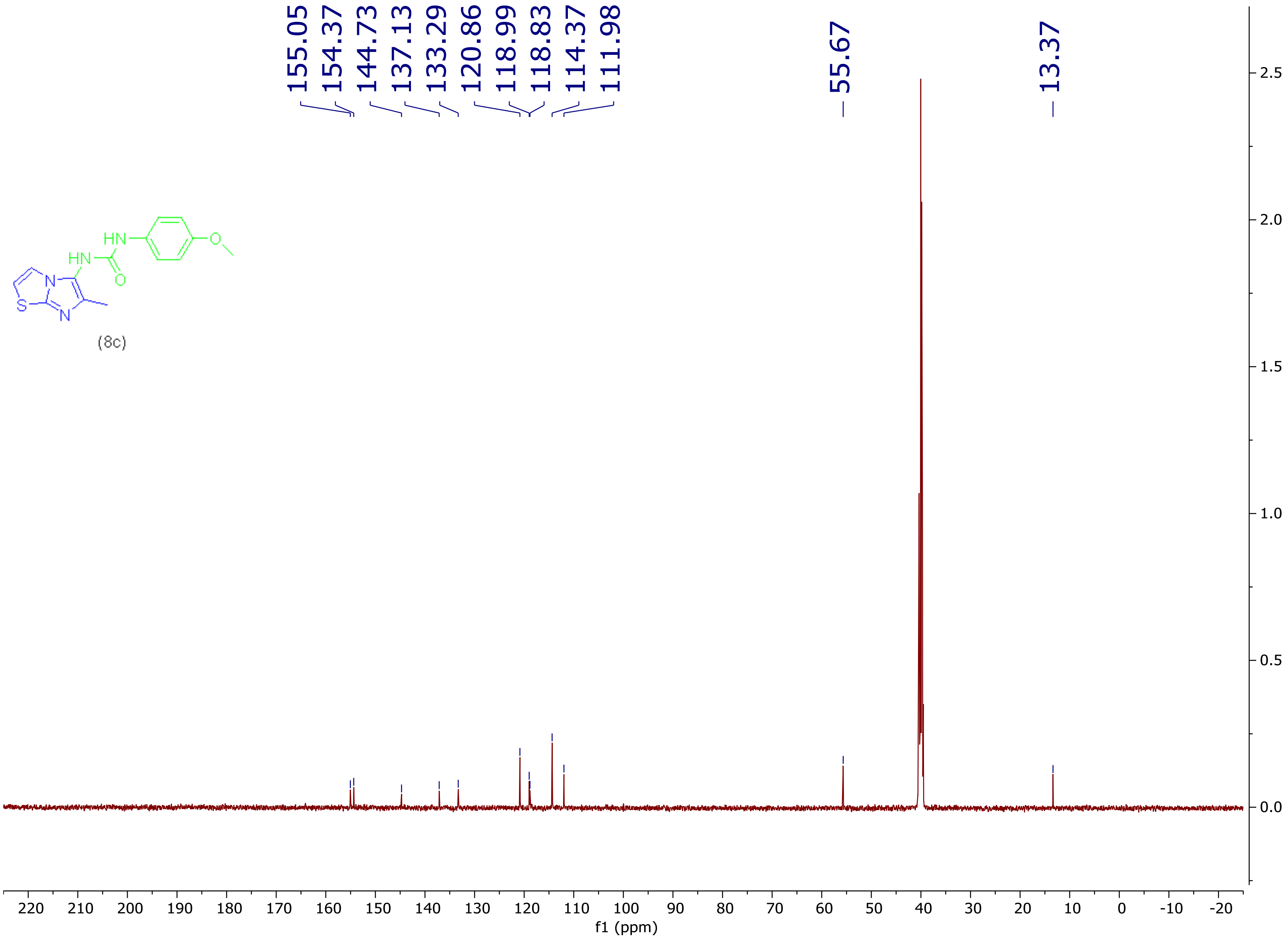

Figure S25. ^{13}C NMR of compound **8c**

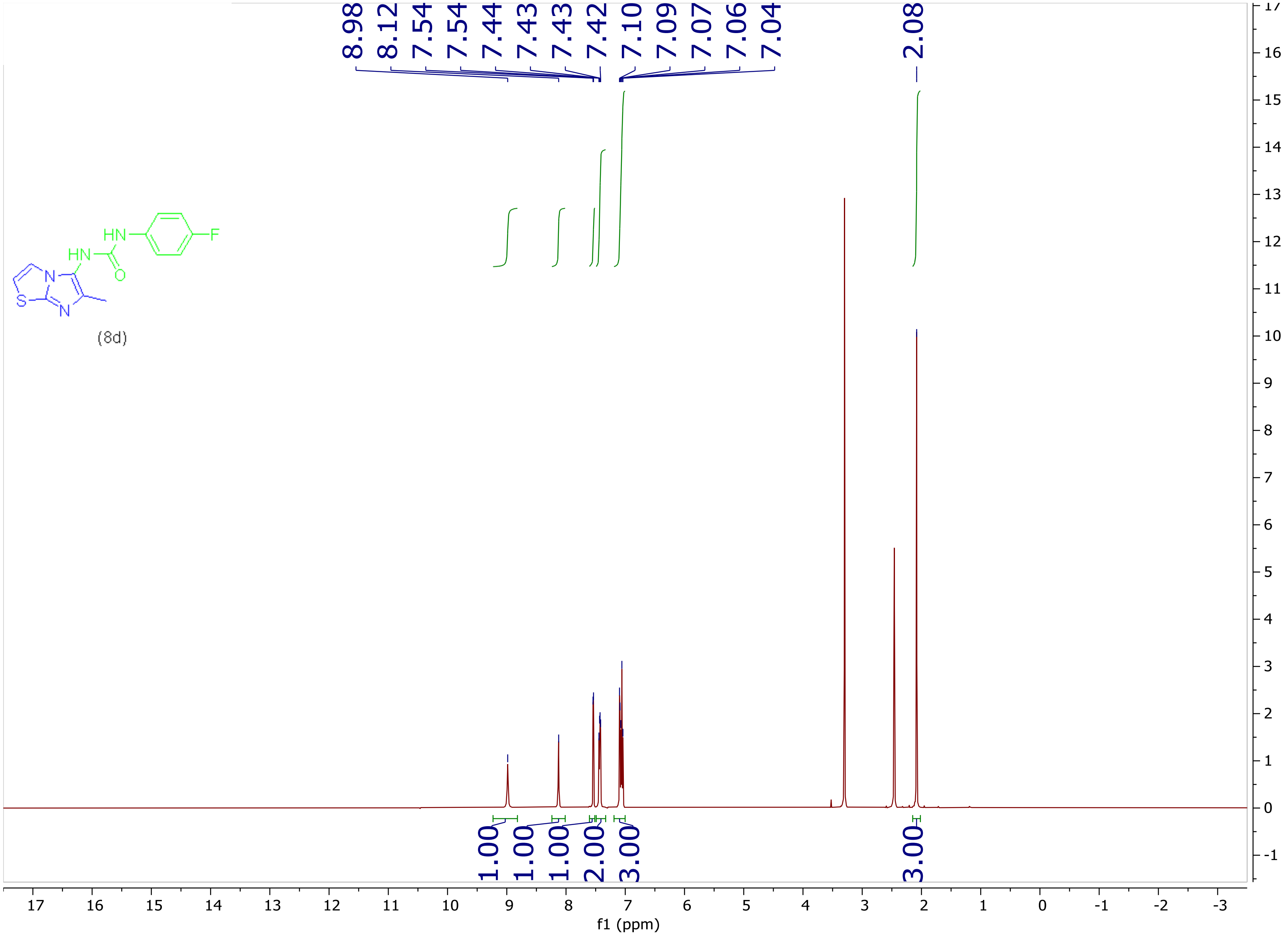

Figure S26. ^{1}H NMR of compound **8d**

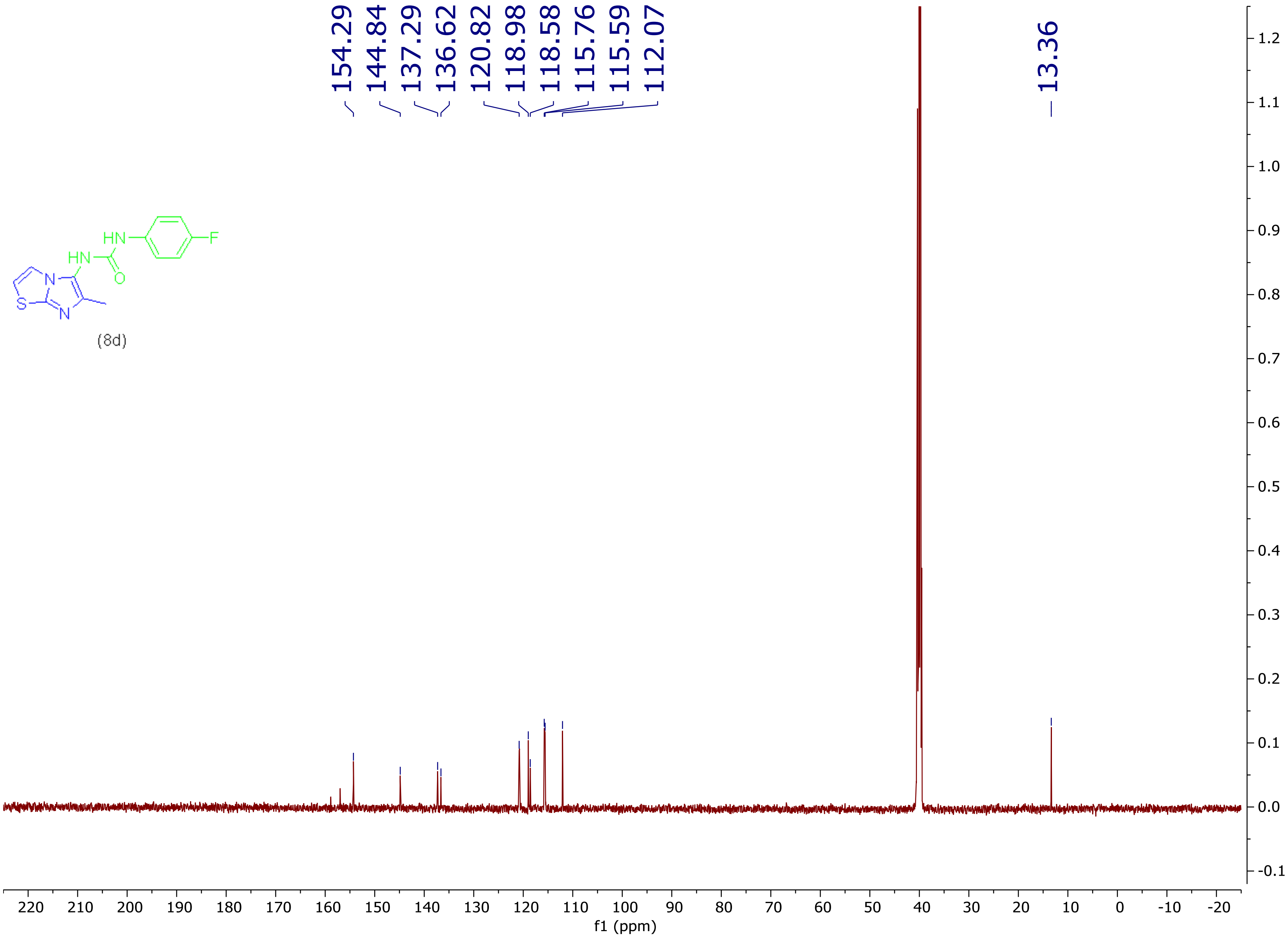

Figure S27. ^{13}C NMR of compound **8d**

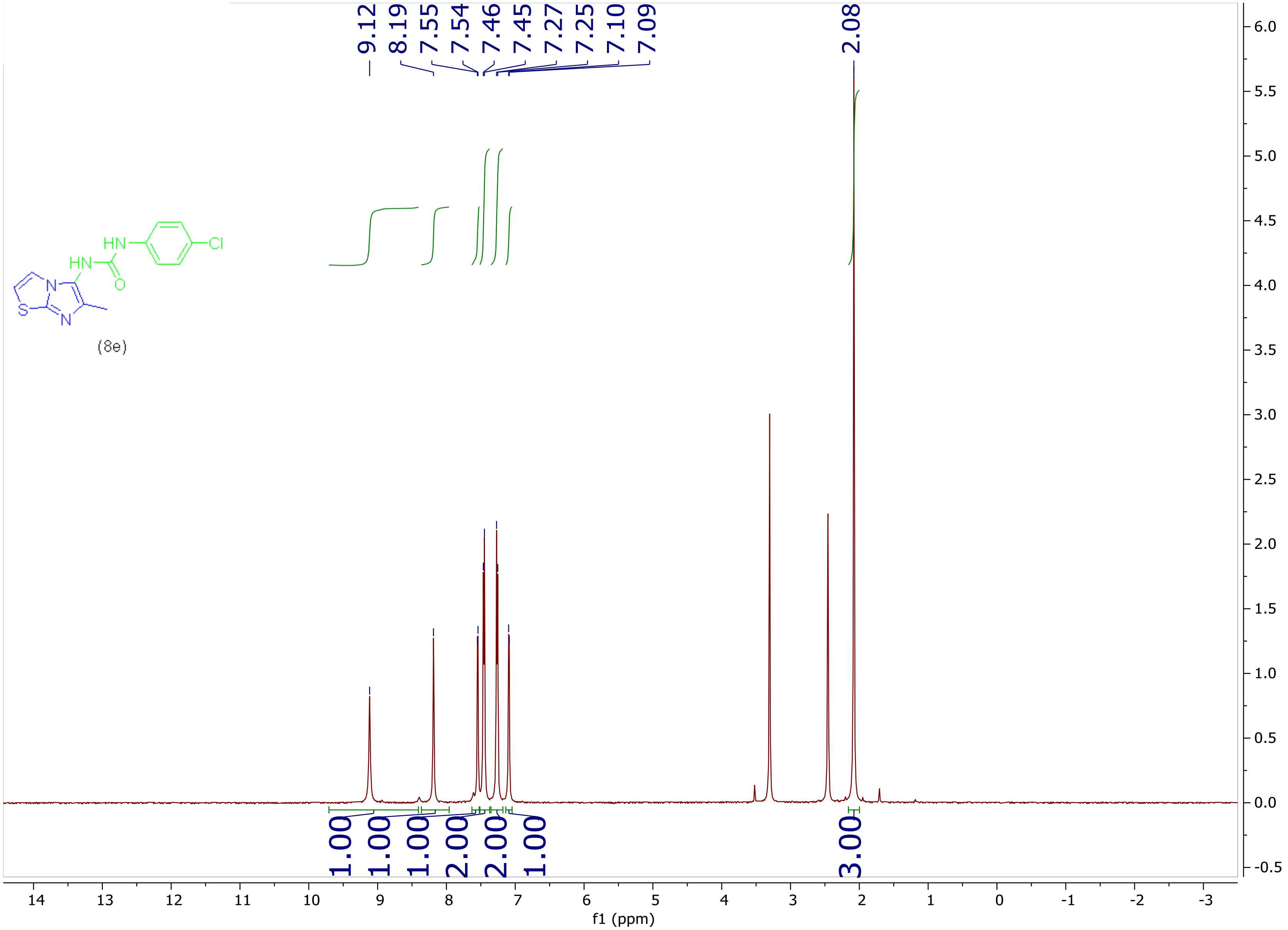

Figure S28. ^{1}H NMR of compound **8e**

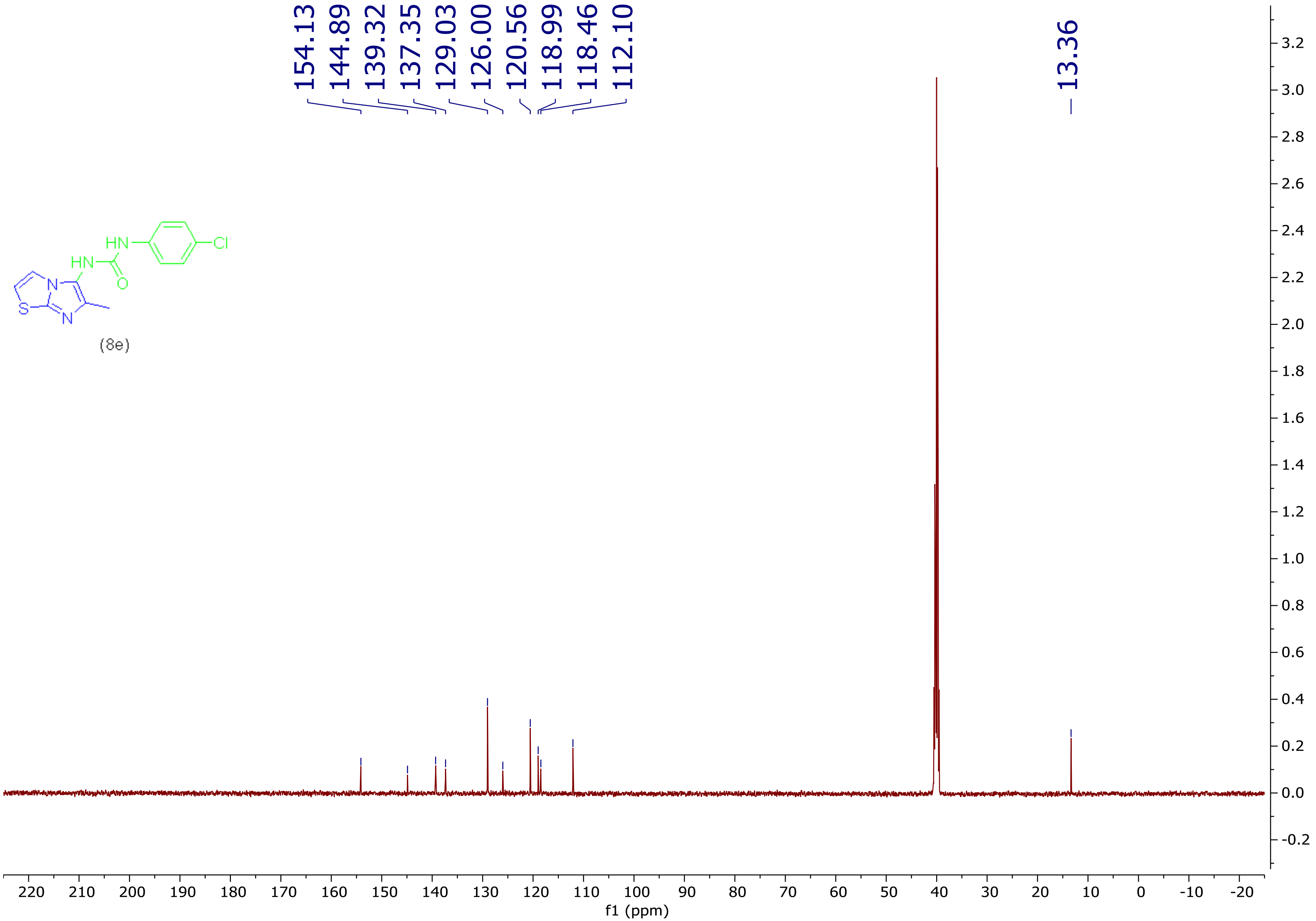

Figure S29. ^{13}C NMR of compound **8e**

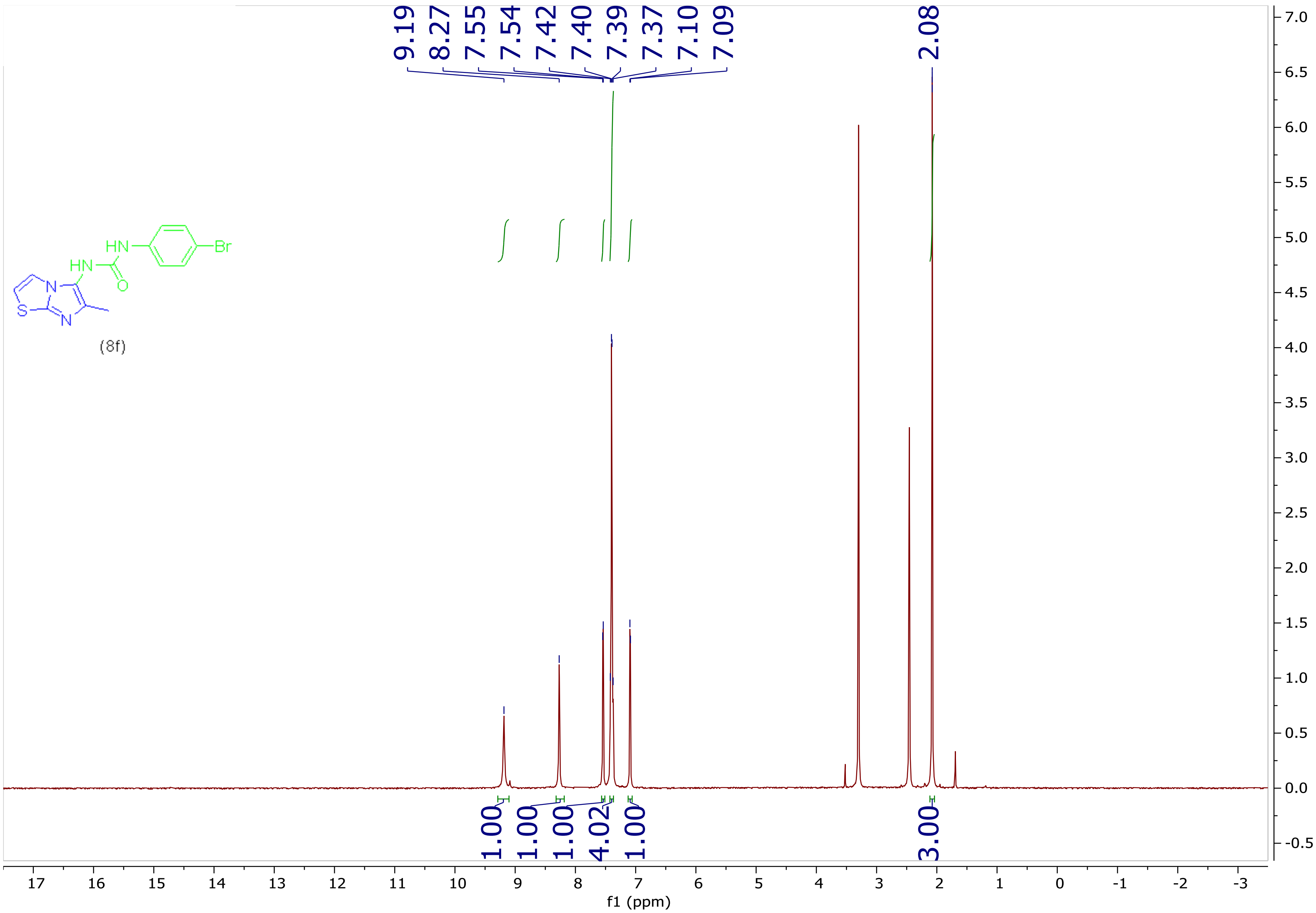

Figure S30. ^{1}H NMR of compound **8f**

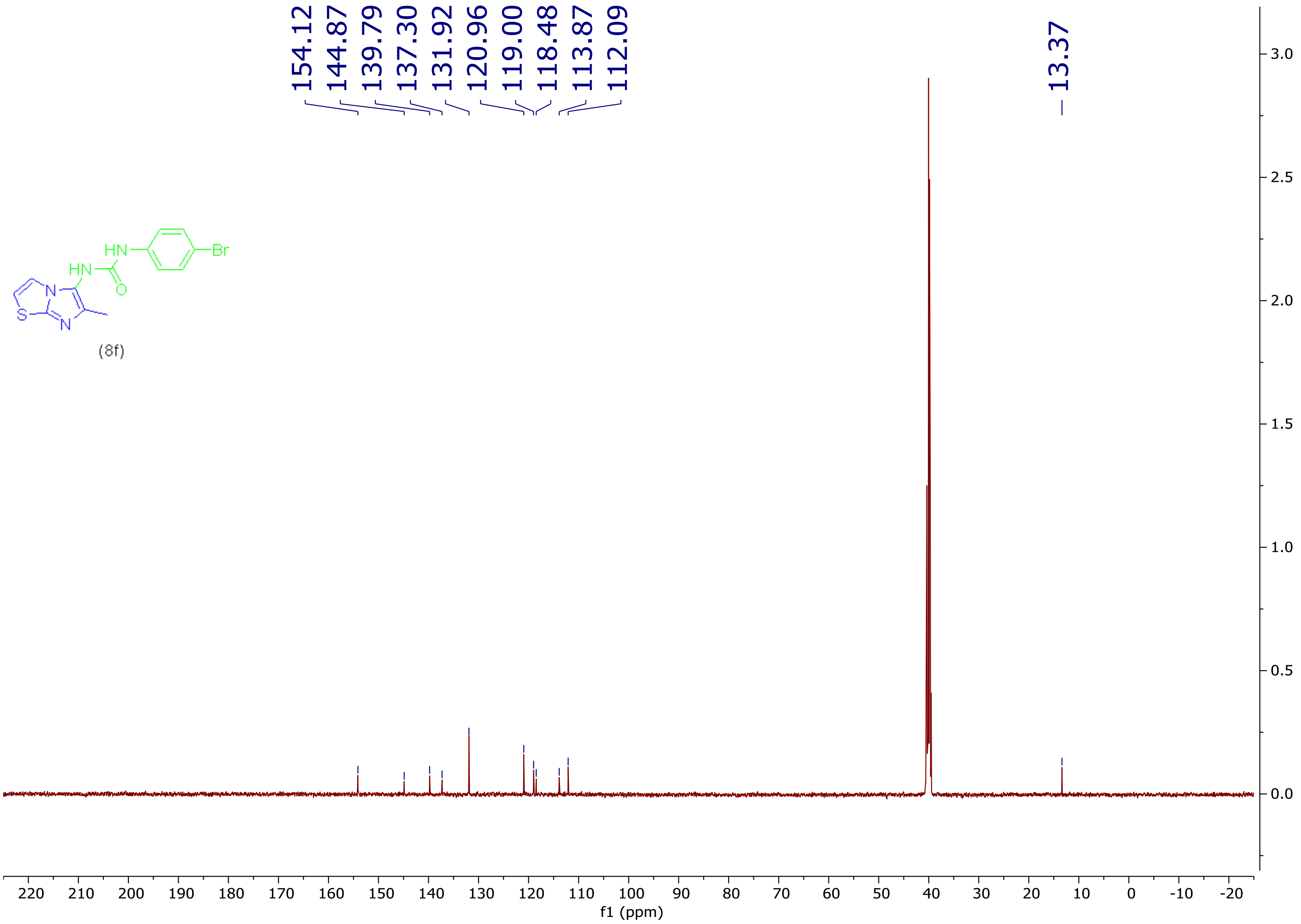

Figure S31. ^{13}C NMR of compound **8f**

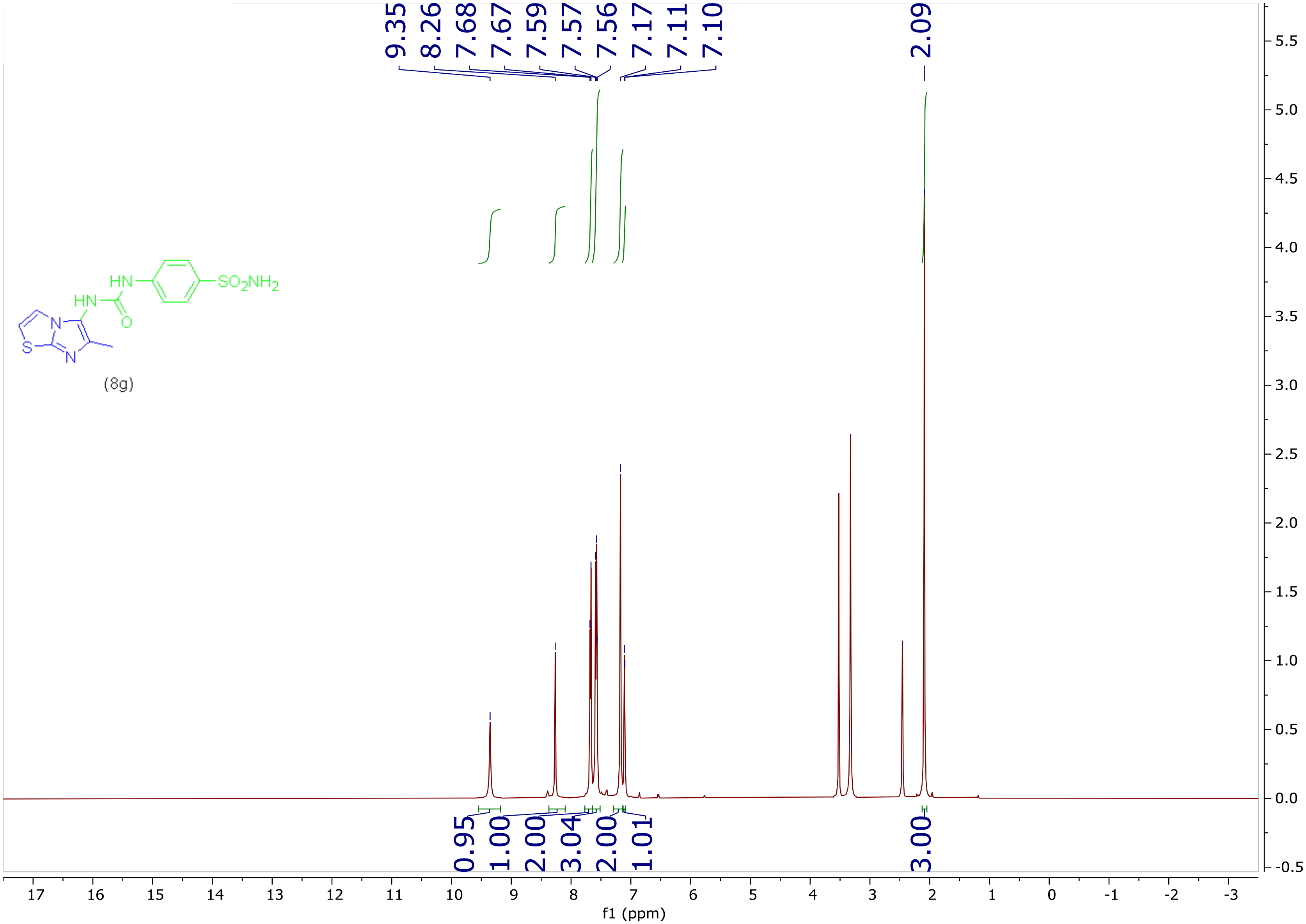

Figure S32. ^{1}H NMR of compound **8g**

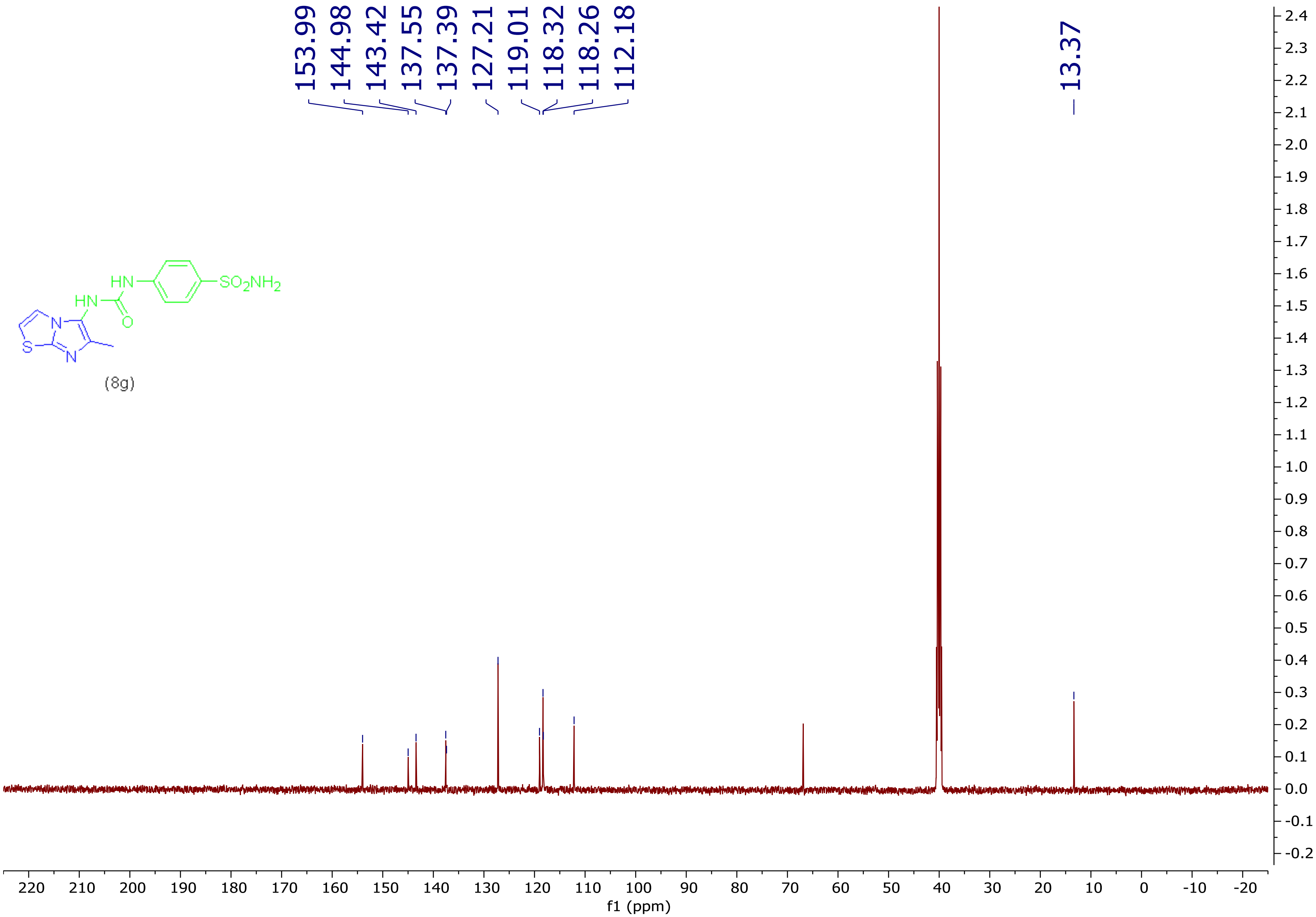

Figure S33. ^{13}C NMR of compound **8g**

Experimental procedures

Chemistry Instruments

- Melting points (°C, uncorrected) were determined using a Stuart melting point apparatus.
- The IR spectra (KBr) ν_{max}/cm^{-1}, were recorded on a SHIMADZU FT/IR spectrometer.
- The NMR spectra in DMSO-d_6, recorded by BRUKER 400 MHz NMR spectrometers. Chemical shifts were reported in parts per million (δ), and coupling constants (J) expressed in Hertz. TMS was used as an internal standard and chemical shifts were measured in δ ppm. ^{1}H and ^{13}C spectra were run at 400 and 100 MHz, respectively. The NH signals were exchanged in D_2O.

MTT assay

The 96 well tissue culture plate was seeded with 1 X 105 cells / ml (100 ul / well) and cultured at 37 degrees Celsius for 24 hours to form a full monolayer sheet. After forming a confluent sheet of cells, growth medium was decanted from 96 well micro titer plates, the cell monolayer was washed twice with wash media, and two-fold dilutions of the tested sample were prepared in RPMI medium with 2% serum (maintenance medium). In each well, 0.1 ml of each dilution was examined, with three wells serving as controls and receiving only maintenance media. The plate was incubated at 37 degrees Celsius and then analyzed. Cells were examined for any physical evidence of toxicity, such as partial or total monolayer loss, rounding, shrinkage, or cell granulation. MTT solution (5mg/ml in PBS) was prepared (BIO BASIC CANADA INC), and 20ul MTT solution was added to each well. Shake the MTT into the medium for 5 minutes at 150rpm on a shaking table. Incubate for 4 hours (37°C, 5% CO2) to allow the MTT to be digested. Remove the media. (If required, dry plate on paper towels to remove residue. In 200ul DMSO, resuspend formazan (MTT metabolic product). Shake at 150rpm for 5 minutes to properly mix the formazan into the solvent. At 560nm, measure optical density and subtract background at 620nm. The optical density should be proportional to the number of cells. MCF-7 and MCF-10A cell lines were provided with 10% fetal bovine serum (FBS, Sigma-Aldrich, MO, USA). Data are presented as the mean of the IC_{50} values ± SD from three different experiments.

VEGF-2 kinase assay

Thaw 5x Kinase Buffer 1, ATP and 50x PTK substrate. (Optional: If desired, add DTT to 5x Kinase Buffer 1 to make a 10 mM concentration; *e.g.* add 10 µl of 1 M DTT to 1 ml 5x Kinase Buffer 1). Prepare the master mixture (25 µl per well): N wells x (6 µl 5x Kinase Buffer 1 + 1 µl ATP (500 µM) + 1 µl 50x PTK substrate + 17 µl water). Add 25 µl to every well. Add 5 µl of

Inhibitor solution of each well labeled as “Test Inhibitor”. For the “Positive Control" and “Blank”, add 5 µl of the same solution without inhibitor (Inhibitor buffer). Prepare 3 ml of 1x Kinase Buffer 1 by mixing 600 µl of 5x Kinase Buffer 1 with 2400 µl water. 3 ml of 1x Kinase Buffer 1 is sufficient for 100 reactions. To the wells designated as "Blank", add 20 µl of 1x Kinase Buffer 1. Thaw VEGFR2 enzyme on ice. Upon first thaw, briefly spin tube containing enzyme to recover full content of the tube. Calculate the amount of VEGFR2 required for the assay and dilute enzyme to 1 ng/µl with 1x Kinase Buffer 1. Store remaining undiluted enzyme in aliquots at -80°C. Note: VEGFR2 enzyme is sensitive to freeze/thaw cycles. Avoid multiple freeze/thaw cycles. Do not re-use thawed aliquots or diluted enzyme. Initiate reaction by adding 20 µl of diluted VEGFR2 enzyme to the wells designated “Positive Control” and "Test Inhibitor Control". Incubate at 30°C for 45 minutes. Data are presented as the mean of the IC_{50} values ± SD from three different experiments.

PCR assay

Incubate complete reaction mix in a real-time thermal detection system as follows: cDNA synthesis: 10 min at 50°C. iScript Reverse transcriptase inactivation: 5 min at 95°C. PCR cycling and detection (30 to 45 cycles): 10 sec at 95°C then 30 sec at 55°C to 60°C (data collection step). *Recommendations for optimal results using the iScript One-Step RT-PCR Kit with SYBR® Green* Primers should be designed according to standard PCR guidelines with a length of 18 to 25 nucleotides, and a GC content of 40% to 65%. Primer design should avoid internal secondary structure, and complementarity at the 3' ends within each primer and primer pair. Optimal results may require titration of primer concentration between 100 and 500 nM. A final concentration of 300 nM per primer is effective for most reactions. In general, reaction efficiency and/or specificity can be optimized using equal concentrations of each primer. For best results, amplicon size should be limited to 50–200 bp. Suggested input quantities of template are: 1 pg to 100 ng total RNA; 10 fg to 100 ng polyA(+) RNA. First strand synthesis can be performed between 40°C and 52°C. Optimal results are generally obtained with a 10-minute incubation at 50°C. Incubation at temperatures higher than 50°C can delay or eliminate the detection of some non-specific amplification artifacts. However, this may also delay the Ct for detection of specific targets. Thaw all components, except the iScript reverse transcriptase, at room temperature. Mix gently, but

thoroughly, and then centrifuge at 4°C to collect contents to the bottom of the tube. Chill on ice before using. Centrifuge again briefly at 4°C if needed. Preparation of a reaction cocktail is crucial in quantitative PCR applications to reduce pipetting errors and maximize assay precision and accuracy. Assemble the reaction cocktail with all required components except sample template (total RNA) and dispense equal aliquots into each reaction tube. Add target sample to each reaction as the final step. Addition of sample as 5–10 µl volumes will improve assay precision. Replicate samples should be assembled as a master mix with a single addition of sample template.

Cell cycle analysis and apoptosis

Transfer the previously prepared cells from 4ºC to the bench-top and equilibrate to room temperature. Gently re-suspend cells by inverting the tube or by gentleup and down pipetting. There may be visible thin salt crystals in the tube but this will not affect the sedimentation of the cells in the next step. Pellet the cells at 500 x g for 5 minutes. Carefully aspirate the supernatant without disrupting the pellet. It is better to incompletely remove the supernatant than to accidently aspirate part of the pellet and lose cells. Wash the cells by gently resuspending in 1 mL 1X PBS. Again, pellet the cells at 500 x g for 5 minutes and carefully remove the supernatant. Gently resuspend the cell pellet in 200 µL 1X Propidium Iodide + RNase Staining Solution. Then ensure that the cells are fully resuspended. Incubate at 37ºC in the dark for 20 – 30 minutes. Place tubes on ice (still in the dark) and prepare for flow cytometry analysis. Gently resuspend cells that settled during the incubation by pipetting up and down. If desired, pass cells through an appropriate filter (not provided) to remove cell aggregates. Run samples on flow cytometer: Set appropriate FSC vs. SSC gates to exclude debris and cell aggregates. Collect Propidium iodide fluorescence in FL2.

Docking Protocol

The Protein Data Bank (http://www.rcsb.org) was utilized to obtain the 3D structures of the protein binding sites of the sorafenib in VEGFR-2 kinase binding site. AutoDockVina 1.5.7 was used to conduct docking studies on established analogues with the VEGFR-2 active site. This program was run with a searching grid that was stretched across ligand molecules, with box spacing of 20×20×20 and x, y, z coordinates of -21.951, -1.151, and -10.205, and all other parameters set to default. The highest scoring position for every compound was chosen after docking it into the crystal structure. The ideal docking posture is thought to be the most stable conformation of every compound attached to the protein-active site. The docked model was chosen

based on docking energies, and the interactions between the ligands and the target were visualized using Discovery studio v21.1.0.20298.

In silico predictive ADME study

Using the SWISSADME server, a free web program developed by the Swiss Institute of Bioinformatics, we evaluated the physicochemical descriptors and projected the pharmacokinetic qualities and druglike characteristics of our target compounds, when we intend to develop novel compounds, it is also critical to consider pharmacokinetic characteristics as well as drug probability properties
